# Spatial and temporal expression of the 23 murine Prolactin/Placental Lactogen-related genes is not associated with their position in the locus

**DOI:** 10.1186/1471-2164-9-352

**Published:** 2008-07-28

**Authors:** David G Simmons, Saara Rawn, Alastair Davies, Martha Hughes, James C Cross

**Affiliations:** 1 Departments of Comparative Biology & Experimental Medicine and Biochemistry & Molecular Biology, The University of Calgary, Calgary, Canada

## Abstract

**Background:**

The Prolactin (*PRL*) hormone gene family shows considerable variation among placental mammals. Whereas there is a single *PRL *gene in humans that is expressed by the pituitary, there are an additional 22 genes in mice including the placental lactogens (PL) and Prolactin-related proteins (PLPs) whose expression is limited to the placenta. To understand the regulation and potential functions of these genes, we conducted a detailed temporal and spatial expression study in the placenta between embryonic days 7.5 and E18.5 in three genetic strains.

**Results:**

Of the 22 *PRL/PL *genes examined, only minor differences were observed among strains of mice. We found that not one family member has the same expression pattern as another when both temporal and spatial data were examined. There was also no correlation in expression between genes that were most closely related or between adjacent genes in the PRL/PL locus. Bioinformatic analysis of upstream regulatory regions identified conserved combinations (modules) of putative transcription factor binding sites shared by genes expressed in the same trophoblast subtype, supporting the notion that local regulatory elements, rather than locus control regions, specify subtype-specific expression. Further diversification in expression was also detected as splice variants for several genes.

**Conclusion:**

In the present study, a detailed temporal and spatial placental expression map was generated for all murine *PRL/PL *family members from E7.5 to E18.5 of gestation in three genetic strains. This detailed analysis uncovered several new markers for some trophoblast cell types that will be useful for future analysis of placental structure in mutant mice with placental phenotypes. More importantly, several main conclusions about regulation of the locus are apparent. First, no two family members have the same expression pattern when both temporal and spatial data are examined. Second, most genes are expressed in multiple trophoblast cell subtypes though none were detected in the chorion, where trophoblast stem cells reside, or in syncytiotrophoblast of the labyrinth layer. Third, bioinformatic comparisons of upstream regulatory regions identified predicted transcription factor binding site modules that are shared by genes expressed in the same trophoblast subtype. Fourth, further diversification of gene products from the *PRL/PL *locus occurs through alternative splice isoforms for several genes.

## Background

The closely related Prolactin (PRL) and growth hormone genes are thought to have arisen from a common ancestral gene as a result of gene duplication and subsequent divergence early in vertebrate evolution [1]. Further gene duplications have given rise to expanded growth hormone or PRL clusters in a species-specific manner. Primates, for example, have an expanded growth hormone locus (containing five genes in close proximity on Ch 17) but a single *PRL *gene. In contrast, rodents have a single growth hormone gene but an amplified *PRL *locus containing 23 *PRL*-like genes in mice [2-4] and at least 25 in the rat [5], all located in close proximity in a single locus. While many of the genes in the mouse and rat *PRL *family are orthologues, some are specific to either species indicating gene duplications since the divergence of mice and rats. Amplification of the *PRL *gene appears to have happened independently at least twice in mammals as ruminants have an expanded *PRL *locus whose members are not orthologous to those of the rodent *PRL *family [3,6-8].

The *PRL *family consists of 23 closely related genes in mice found within a one megabase locus on chromosome 13 [2-4]. PRL was originally identified as a pituitary hormone involved in mammary gland development [9] but is now understood to have a wide range of biological actions and targets. PRL is of particular importance for female reproduction and mice harboring null mutations in the PRL or PRL receptor genes have deficits in ovarian hormone production, decidualization, pup-induced maternal behavior and other adaptations to pregnancy [10-12]. The mammalian placenta is an important endocrine organ coordinating maternal and fetal responses to pregnancy and is a source of extrapituitary lactogenic activity as it produces several lactogenic PRL-related proteins. The first PRL-related proteins were discovered in rodents and called Placental Lactogen I and II (PL-I and PL-II) based on their lactogenic effects. With molecular cloning and genome sequencing, the family has grown to include at least three genes encoding PL-I proteins in mice plus many other more distantly related genes, although not all family members are lactogenic. All of the PRL/PL-related genes are expressed at the fetal maternal interface, predominantly in cells of the trophoblast lineage [3,4]. The nomenclature of the *PRL/PL *genes has recently been revised [13] and the new nomenclature will be used throughout the paper (see Table 1).

**Table 1:** Standardized nomenclature for the mouse PRL/PL family

PRL Family Gene Name	Official Symbol	Official Alias Symbol	Mouse Genbank Accession No.
Prolactin	Prl	Prl1a1	NM_011164
Prolactin family 2, subfamily a, member 1	Prl2a1	PLP-M	NM_019991
Prolactin family 2, subfamily b, member 1	Prl2b1	PLP-K	NM_025532
Prolactin family 2, subfamily c, member 2	Prl2c2	PLF1	NM_031191
Prolactin family 2, subfamily c, member 3	Prl2c3	PLF2	K03235
Prolactin family 2, subfamily c, member 4	Prl2c4	MRP3	NM_011954
Prolactin family 2, subfamily c, member 5	Prl2c5	MRP4	AF128884
Prolactin family 3, subfamily a, member 1	Prl3a1	PLP-I	AF525154
Prolactin family 3, subfamily b, member 1	Prl3b1	PL-II, Csh2	M14647
Prolactin family 3, subfamily c, member 1	Prl3c1	PLP-J	NM_013766
Prolactin family 3, subfamily d, member 1	Prl3d1	PL-Iα, Csh1α	AF525162
Prolactin family 3, subfamily d, member 2	Prl3d2	PL-Iβ, Csh1β	NM_172155
Prolactin family 3, subfamily d, member 3	Prl3d3	PL-Iγ, Csh1γ	NM_172156
Prolactin family 4, subfamily a, member 1	Prl4a1	PLP-A	NM_011165
Prolactin family 5, subfamily a, member 1	Prl5a1	PLP-L	NM_023746
Prolactin family 6, subfamily a, member 1	Prl6a1	PLP-B	NM_011166
Prolactin family 7, subfamily a, member 1	Prl7a1	PLP-E	NM_008930
Prolactin family 7, subfamily a, member 2	Prl7a2	PLP-F	NM_011168
Prolactin family 7, subfamily b, member 1	Prl7b1	PLP-N	AF525156
Prolactin family 7, subfamily c, member 1	Prl7c1	PLP-O	NM_026206
Prolactin family 7, subfamily d, member 1	Prl7d1	PLF-RP	NM_011120
Prolactin family 8, subfamily a, member 1	Prl8a1	PLP-Cδ	NM_028477
Prolactin family 8, subfamily a, member 2	Prl8a2	dPRP	NM_010088
Prolactin family 8, subfamily a, member 6	Prl8a6	PLP-Cα	NM_011167
Prolactin family 8, subfamily a, member 8	Prl8a8	PLP-Cγ	NM_023741
Prolactin family 8, subfamily a, member 9	Prl8a9	PLP-Cβ	NM_023332

The Placental Lactogen proteins (now called *Prl3b *and *Prl3d*) bind to the PRL receptor and mimic the actions of pituitary PRL [14], which include multiple pregnancy-dependent processes such as mammary gland development, corpus luteum function [15], maternal behavior [16] and pancreas function [17]. However, the additional PRL/PL-related proteins are 'non-classical' members of the PRL family as they do not bind and activate the PRL receptor, although receptor-binding activity for several members has not yet been investigated. Overall, the biological targets and functions are known for only a few of the PRL/PL-related proteins. Prl2c (Proliferin) [18] and Prl7d1 (Proliferin-related protein) [19] have angiogenic and anti-angiogenic effects on endothelial cells, respectively [20]. Prl7a1 (PLP-E) and Prl7a2 (PLP-F) promote blood cell production [21,22]. Prl4a1 (PLP-A) suppresses natural killer cells [23]. The only mouse mutants that have been reported so far are for Prl4a1 [24] and Prl8a2 (dPRP) [25]. Neither mouse mutant shows an obvious phenotype under normal conditions but embryonic lethality ensues when pregnant mice are exposed to hypoxia. This implies either that the *PRL/PL*-related genes have evolved to respond to physiological stress or alternatively that redundancy within the gene family minimizes the effects of single gene mutations.

The human growth hormone locus relies on a locus control region 15–32 kilobases upstream to drive tissue-specific expression [26,27]. Within the *PRL/PL *locus in rodents, however, there is no evidence so far for similar control of regulation and no correlation between gene location within the *PRL/PL *locus and temporal or spatial expression patterns within the placenta has been observed [4]. Placental expression data for most of the murine *PRL *family members has been reported [4,25,28-34], although almost exclusively at mid-gestation, and comprehensive temporal and spatial expression data for all 23 members of the PRL/PL family has not yet been compiled. We have recently expanded on trophoblast cell type classifications within the murine placenta [35]. Our objective here was to generate a high-resolution map of *PRL/PL *expression patterns and explore associations between co-expressed genes and their respective positions within the locus and/or similarities in regulatory elements.

## Methods

### cDNA probes

cDNAs with the full length open reading frames for each *PRL/PL *family member were used as probes for northern blot and in situ hybridization. cDNAs for *Prl3b1*, *Prl2c1*, *Prl7d1 *were kindly provided by Dr. J. Rossant. cDNAs for *Prl3d1*, *Prl8a2*, *Prl4a1*, *Prl6a1*, *Prl7a1*, *Prl3c1*, *Prl5a1 *were kindly provided by Dr. D. Linzer. cDNAs for *Prl8a9*, *Prl8a8*, *Prl3a1*, *Prl2b1*, *Prl2a1*, *Prl7b1*, *Prl7c1 *were kindly provided by Dr. M. Soares. Full length cDNAs for *Prl*, *Prl8a6*, *Prl8a1*, *Prl7a2*, and *Pcdh12 *were generated by PCR: *Prl *(forward 5'-CTCTCAGGCCATCTTGGAGA-3', reverse 5'-TAAGCAGTTGTTTTGATGGGC-3); *Prl8a6 *(forward 5'-ACGATGGCACTGCTATTGAGT-3', reverse 5'-TGGAGCATTTTCAAAGCAGA-3'); *Prl8a1 *(forward 5'-CAAAGATGGTGCTGCCATTA-3', reverse 5'-CCCAGTTATGCGACATTTCA-3'); *Prl7a2 *(forward 5'-GCCAGAACTCTTCAGAGATGTC-3', reverse 5'-TTAAGCATCATGAAGCATCTCT3'); *Pcdh12 *(forward 5'-CAATGGGAATCCCCCTAAGT-3', reverse 5'-TAGGTGGTCCACACTGGTGA-3'). PCR fragments were cloned into pGEM-T easy (Promega) and sequence verified.

### PCR primers and conditions

RT-PCR was used to determine whether predicted isoforms of *Prl6a1*, *Prl8a6*, *Prl8a8*, *Prl8a1*, and *Prl7a1 *were expressed in vivo. RNA was isolated from implantation sites or placentas at various stages of gestation by using Trizol (Invitrogen) according to the manufacturer's instructions. cDNA synthesis employed oligo dT primer and superscript enzyme (Invitrogen) according to the manufacturer's instructions. PCR primer sets and conditions are listed in Table 2. All PCR amplicons were cloned into pGEM-T easy (Promega) and sequence verified.

**Table 2:** Primers and conditions for isoform-specific PCR analysis

PCR Primer Name	Primer Sequence	PCR Conditions
*Prl6a1 *forward	5'-GGTCATTTCCCAGATGCTGT-3'	1.5 mM MgCl_2_, 35 cycles, 60°C annealing
*Prl6a1 *common reverse	5'-GGGCTCCCTCCTTAGACACT-3'	
*Prl6a1 *novel forward	5'-GCCTGAGAAGGCAAAGAAAA-3	

*Prl8a6 *forward	5'-CTGCCATTCAAGTCTCACGA-3'	1.5 mM MgCl_2_, 30 cycles, 60°C annealing (Novel forward rxn – 35 cycles)
*Prl8a6 *common reverse	5'-CTCAAAAGCCAAGGAGATCG-3'	
*Prl8a6 *novel forward	5'-GCTCGATTATATAGGAACAGGAA-3'	

*Prl8a8 *forward	5'-GAAAAAGAATTGTGATT-3'	2.0 mM MgCl_2_, 35 cycles, 46°C annealing (Novel forward rxn – 1.5 mM MgCl_2_)
*Prl8a8 *common reverse	5'-TCAATAGTTATGTATTGAA-3'	
*Prl8a8 *novel forward	5'-ATAACTCCGATGAAGC-3'	

*Prl8a1 *reverse	5'-CCAAACAATCAACTGCCATG-3'	1.5 mM MgCl_2_, 30 cycles, 60°C annealing
*Prl8a1 *common forward	5'-AACCCCACTTCTGCACATTC-3'	
*Prl8a1 *novel reverse	5'-CCCAAAAGATCGACCCATAA-3'	

*Prl7a1 *reverse	5'-TCAGCCACATTTTCTGTTGC-3'	2.0 mM MgCl_2_, 28 cycles, 60°C annealing (Novel reverse rxn 32 cycles)
*Prl7a1 *common forward	5'-AAGGGACTGTTGGATCATGC-3'	
*Prl7a1 *novel reverse	5'-TGGCATTCATGAGTGAGAAA-3'	

### Phylogenetic analysis and transcription factor binding site analysis

cDNA sequences of the 23 paralogous *PRL/PL *family members were input into the Mega 3.1 software program to generate a phylogenetic tree [36]. Three kilobases of upstream promoter regions of paralogous *PRL/PL *family members were analyzed for transcription factor binding sites by using the Toucan 2 software program [37].

### Northern blot analysis

Northern blot analysis was performed as previously described [35]. Briefly, RNA was extracted by using Trizol (Invitrogen), separated on a 1.1% formaldehyde gel and transferred to nylon membranes (Perkin Elmer). After UV cross-linking, the membranes were hybridized in Church buffer [38] containing ^32^P-labeled cDNA probes. An 18S ribosomal RNA probe was used to check for RNA integrity and standardize for gel loading.

### In situ hybridization

Placentae were dissected in cold phosphate buffered saline (PBS) and fixed overnight in 4% paraformaldehyde in PBS at 4°C. After rinsing in PBS, tissues were processed through sucrose gradients (10% in PBS and 25% in PBS) before being embedded in OCT (Tissue Tek). Ten μm sections were cut and mounted on Super Frost Plus slides (VWR) and stored at -80°C. For in situ hybridization, sections were re-hydrated in PBS, post-fixed in 4% paraformaldehyde for 10 minutes, treated with proteinase K (30 μg/ml) for 10 minutes at room temperature, acetylated for 10 minutes (acetic anhydride, 0.25%; Sigma) and hybridized with digoxigenin-labeled probes overnight at 65°C. Digoxigenin labeling was done according to the manufacturers instructions (Roche). Hybridization buffer contained 1× salts (200 mM sodium choride, 13 mM tris, 5 mM sodium phosphate monobasic, 5 mM sodium phosphate dibasic, 5 mM EDTA), 50% formamide, 10% dextran sulfate, 1 mg/ml yeast tRNA (Roche), 1× Denhardt's (1% w/v bovine serum albumin, 1% w/v Ficoll, 1% w/v polyvinylpyrrolidone), and DIG-labeled probe (final dilution of 1:2000 from reaction with 1 μg template DNA). Two 65°C post-hybridization washes (1× SSC, 50% formamide, 0.1% tween-20) followed by two room temperature washes in 1× MABT (150 mM sodium chloride, 100 mM maleic acid, 0.1% tween-20, pH7.5) were followed by 30 minutes RNAse treatment (400 mM sodium chloride, 10 mM tris pH7.5, 5 mM EDTA, 20 μg/ml RNAse A). Sections were blocked in 1× MABT, 2% blocking reagent (Roche), 20% heat inactivated goat serum for 1 hour and incubated overnight in block with anti-DIG antibody (Roche) at a 1:2500 dilution at 4°C. After four 20 minute washes in 1× MABT, slides were rinsed in 1× NTMT (100 mM NaCl, 50 mM MgCl, 100 mM tris pH 9.5, 0.1% tween-20) and incubated in NBT/BCIP in NTMT according to the manufacturers instructions (Promega). Slides were counterstained with nuclear fast red, dehydrated and cleared in xylene and mounted in cytoseal mounting medium (VWR).

For double labeled in situ hybridizations, a fluorescein-labeled probe was generated following the manufacturers instructions (Roche) and added to the hybridization mix along with the DIG-labeled probe. Following NBT/BCIP development of the first probe, the antibody-enzyme conjugate was inactivated by 30 min incubation in 1× MABT at 65°C, followed by 30 min in 0.1 M glycine pH 2.2 at room temperature. The slides were then blocked again in 1× MABT, 2% blocking reagent (Roche), 20% heat inactivated goat serum for 1 hour and incubated overnight in block with anti-Fluorescein antibody (Roche) at a 1:2500 dilution at 4°C. The second signal was developed as previously described but one modification, the substrate INT/BCIP (Roche), producing a brown colour, was used in place of NBT/BCIP. As the INT/BCIP is soluble in alcohols and xylene, slides were counterstained with nuclear fast red and mounted directly under Crystal Mount Aqueous Mounting Medium (Sigma).

## Results

### PRL/PL Family Nomenclature and Expression Analysis

A revised system for naming members of the mouse and rat *PRL/PL *gene families has recently been introduced [13] and is used throughout (see Table 1). Our analysis was aimed at being able to discriminate the patterns of expression of all 23 genes in the *PRL/PL *locus. However, the similarity of some family members precluded us form being able to do this for both spatial and temporal analysis. For example, the new nomenclature cites four additional murine *Prl2c *(*Proliferin*) genes (*Prl2c2, Prl2c3, Prl2c4, and Prl2c5*) since four cDNAs have been reported, but the actual number of genes and their respective locations within the genome remain a matter of debate. Differentiation among the various *Prl2c *cDNAs requires RT-PCR analysis followed by restriction enzyme digestion of the resultant amplicons [39-41]. Therefore, as the methodology used in the current study (cDNA probe for *Prl2c1 *used in northern blot analysis and in situ hybridization) could not discriminate expression from the various *Prl2c *genes or isoforms, hypothesized or annotated, the data for all genes was summarized as *Prl2c*. Similarly, data for *Prl3d1*, *Prl3d2 *and *Prl3d3 *(*Placental Lactogen I alpha*, *beta *and *gamma*) are summarized as *Prl3d*.

**Figure 1:**
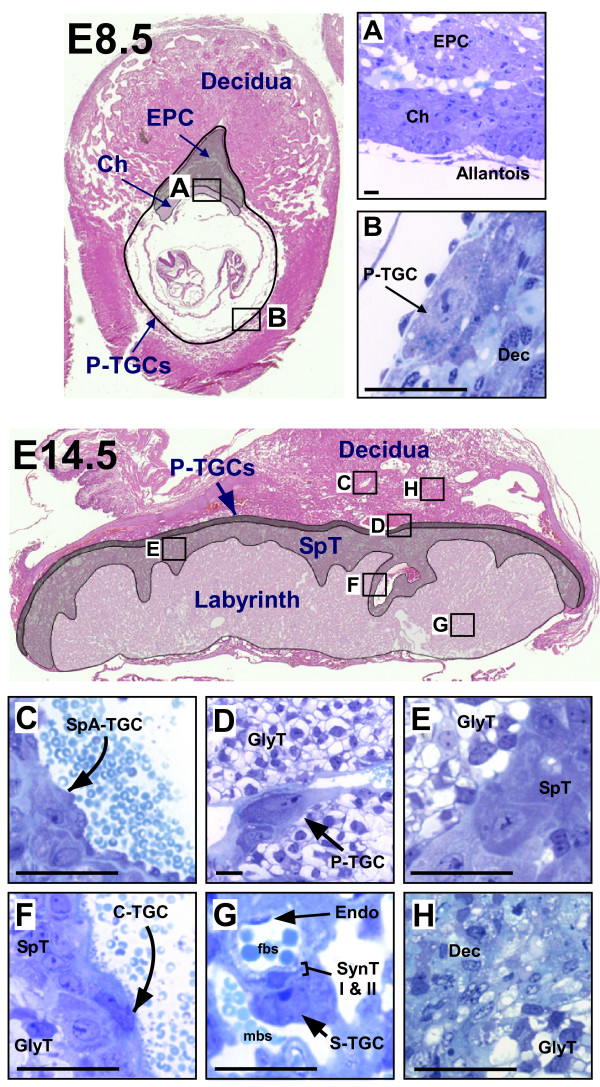
**Placental anatomy and trophoblast subtypes at early and mid-gestation**. A, B. Embryonic day 8.5 implantation site. A – The collapse of the ectoplacental cone cavity (~E8.0) brings the base of the ectoplacental cone into contact with the distal surface of the chorion, while the allantois makes contact with, and adheres to, the basal surface of the chorion by E8.5. B – Large parietal TGC cells line the implantationsite. C-H. Embryonic day 14.5 placenta. C – Spiral artery lined by spiral artery TGCs. D – parietal TGC with glycogen trophoblast cells located beneath within the spongiotrophoblast layer as well as glycogen trophoblast cells which have invaded above the parietal TGC layer into the decidua. E – Clusters of glycogen trophoblast cells within the spongiotrophoblastlayer. F – Large central canal lined by canal TGCs. G – The maternal blood sinuses and fetal blood vessels (lined by endothelial cells) of the labyrinth are separated by a trilaminar trophoblast layer; sinusoidal TGCs that line the maternal sinusoids and two layers of syncytiotrophoblast. H – Clusters of glycogen trophoblast cells invade into the decidua beginning on E12.5. Ch – chorion, C-TGC – canal trophoblast giant cell, Dec – decidua, EPC – ectoplacental cone, Endo – endothelial cell, GlyT – glycogen trophoblast cell, P-TGC – parietal trophoblast giant cell, S-TGC – sinusoidal trophoblast giant cell, SpA-TGC – spiral-associated trophoblast giant cell, SpT – spongiotrophoblast, SynT – syncytiotrophoblast cell (I – layer I, II- layer II). Black bar represents 0.1 mm.

In situ hybridization was used to define the spatial patterns of expression between embryonic days (E) 8.5 and 18.5 (term gestation). The various trophoblast cell types in the mouse placenta are shown in Figure 1 for reference. In general, the fully developed placenta has distinct layers: a maternal decidual zone which also contains spiral artery-associated trophoblast giant cells (TGC) and glycogen trophoblast cells that invade into it; a junctional zone consisting of parietal TGCs, spongiotrophoblast cells and glycogen trophoblast cells; and finally the labyrinth, in which TGCs line maternal blood canals that enter the labyrinth leading into small sinusoids lined by sinusoidal TGCs and syncytiotrophoblast cells (Fig 1) [35,42,43]. Northern blotting of whole placental tissue was used to confirm the specificity of the cDNA probes and to assess temporal expression. Analysis was performed in three commonly used mouse strain backgrounds (CD1 (Charles River) or Swiss, C57/B6, 129 svj), which are independently derived strains, to explore possible variation.

### Expression Profiles for PRL/PL Family Members Throughout Placental Development

**Figure 2:**
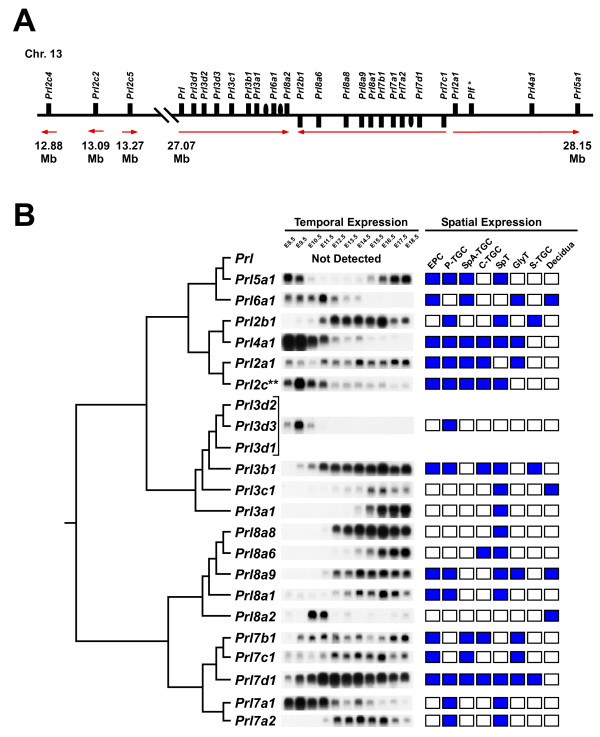
**Temporal and spatial expression profiles of PRL/PL family members compared with phylogenetic tree analysis**. A – Scale diagram of the PRL/PL gene locus on chromosome 13. Rectangles indicate the location of PRL family member genes and ovals represent the location of pseudogenes within the locus. Red arrows indicate the orientation of each gene within the cluster. Note the large inversion from *Prl2b1 *to *Prl7c1*. In addition, three *Prl2c *genes are located approximately 14 Mb upstream of the PRL/PL locus on chromosome 13. *The *Prl2c *gene located within the main prolactin family cluster (27–28 Mb) does not correspond to any of the 4 *Prl2c *genes previously annotated from cDNA sequences (*Prl2c2-5*) and is therefore referred to as *Plf*, the original gene symbol. B – Phylogenetic tree depicting the evolutionary relationship between the members of the PRL family coupled with temporal (northern blot analysis) and spatial (in situ hybridization summary) expression data for each gene. Blue boxes indicate some positive expression within the trophoblast subtype population while white boxes represent an absence of gene expression. **As the differences between the *Prl2c *genes (*Prl2c2-5*) cannot be discerned by northern blot analysis, the results in the current analysis reflect the combined expression of all these genes, and is therefore labeled simply as *Prl2c*.

The summary of northern blot and in situ hybridization analysis is shown in Figure 2 and Additional file 1, and the detailed data for each gene is presented in Additional files 2, 3, 4, 5, 6, 7, 8, 9, 10, 11, 12, 13, 14, 15, 16, 17, 18, 19, 20, 21, (see additional files 2, 3, 4, 5, 6, 7, 8, 9, 10, 11, 12, 13, 14, 15, 16, 17, 18, 19, 20, 21). *Prl1a1 *(*Prl*) expression was not detected in the placenta whereas all of the other genes were, in distinct patterns. The expression patterns were generally consistent across the three strains and data are shown for the CD1 background. However, a subset of *PRL/PL*-related genes displayed subtle variations by strain, either in the timing of the onset/offset of expression within a particular trophoblast subtype or in the number of cells of a given subtype that express the *PRL *family member. These small differences are outlined in Additional files 3, 11, 16 and 20. Unexpected and unpublished expression patterns were also noted, particularly the expression of many *PRL/PL *genes in the developing ectoplacental cone (see below) and the segregation of certain family members to the spongiotrophoblast or glycogen cell populations within the junctional zone (discussed below). Interestingly, not one *PRL/PL *gene was expressed in the chorion early in placental development or in the syncytiotrophoblast cells of the mature labyrinth.

In general, because of the inclusion of numerous gestational time points and detailed spatial analysis, our analysis generated new expression information for all family members. However, three family members showed discrepancies with previously published data. *Prl3c1 *(Prolactin-like protein J) is expressed in the antimesometrial decidua early in pregnancy and lasts as long as the antimesometrial decidual tissue persists [25,31]. In the current study, *Prl3c1 *expression was also detected in the mesometrial decidua, although to a lesser extent, and in spongiotrophoblast at mid-gestation (see Additional file 3). *Prl8a1 *(Prolactin-like protein C delta) expression was not detected in the migratory glycogen trophoblast cells at mid-gestation (see Additional file 12), or as strongly in the spongiotrophoblast cells compared to a previous report [4]. Finally, *Prl7c1 *(Prolactin-like protein O) has been reported to be expressed in spongiotrophoblast, TGCs and the labyrinth [4] but in the present study *Prl7c1 *expression was detected in the ectoplacental cone early in gestation and was then restricted to glycogen trophoblast cells later in gestation, although by E12.5 expression was very much reduced (see Additional file 17). Some of these differences could be explained by differences in the use of in situ hybridization compared with immunohistochemistry or with the use of different in situ hybridization probes or sensitivity, but the significant discrepancy regarding *Prl7c1 *expression is more difficult to reconcile. We have re-sequenced all of our cDNA plasmids to confirm that we used the correct probes.

### No Correlation Between the Spatial/Temporal Expression Profiles of PRL/PL Family Members and their Position within the Locus or Sequence Similarity

In comparing the expression profiles of the genes in the *PRL/PL *locus, each gene had a largely unique pattern when considering both spatial and temporal expression. By aligning the expression patterns with an evolutionary tree based on gene sequence similarity (Fig. 2B) or with position along the locus (see Additional file 1), it was clear that there were no obvious patterns. This was true regardless of the phylogenetic methodology applied or the nature of the comparison (cDNA versus amino acid sequence).

### Expression of Numerous Prolactin Family Members within the Ectoplacental Cone

**Figure 3:**
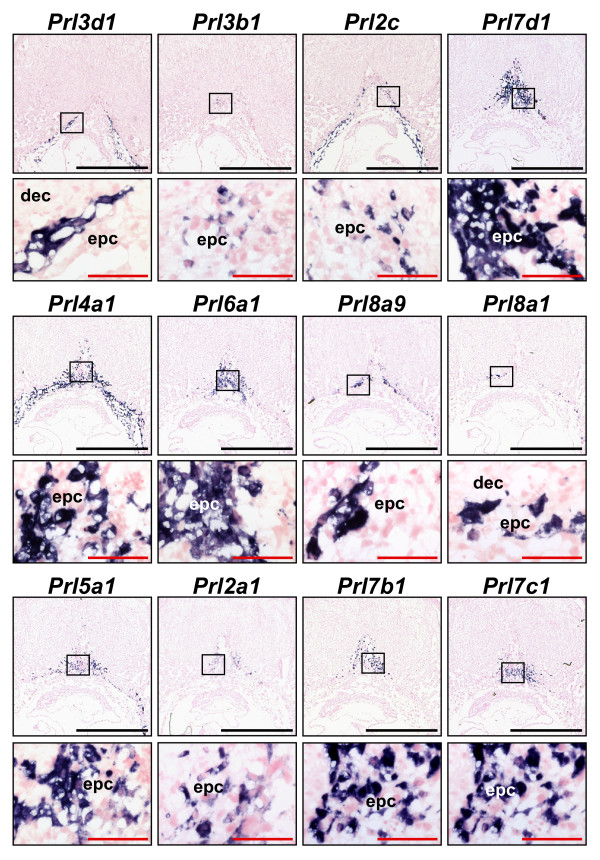
**Expression of numerous PRL/PL family members within the ectoplacental cone**. In situ hybridization of *PRL*/*PL *family members in E8.5 implantations sites. Black boxes represent the area magnified in the image below. Dec – decidua, EPC – ectoplacental cone. Black bar represents 1.5 mm. Red bar represents 100 μm.

An unexpected finding was that expression of at least half of the *PRL/PL *family members was detected within the ectoplacental cone at E8.5 (Figs 2 and 3). *Prl3d1*, *Prl2c*, *Prl7a1 *(not shown, same pattern as *Prl3d1*, see Additional file 14), *Prl4a1*, *Prl8a9*, *Prl8a1 *and *Prl5a1 *were detectable in parietal TGCs at the periphery of the ectoplacental cone at E8.5, although not all were uniformly expressed in all parietal TGC (Fig 3). Only *Prl3d *and *Prl7a1 *were truly restricted to the parietal TGC subtype however, as *Prl2c*, *Prl4a1*, *Prl8a1 *and *Prl5a1 *were also expressed with the core of the EPC (Fig 3). In contrast, expression of *Prl3b1*, *Prl7d1*, *Prl6a1*, *Prl2a1*, *Prl7b1 *and *Prl7c1 *were detected within the centre of the ectoplacental cone and not in parietal TGCs (Fig 3). Again, however, not all the expression patterns within the ectoplacental cone appeared to be uniform or similar to each other.

### Glycogen Trophoblast or Spongiotrophoblast-Specific Expression

**Figure 4:**
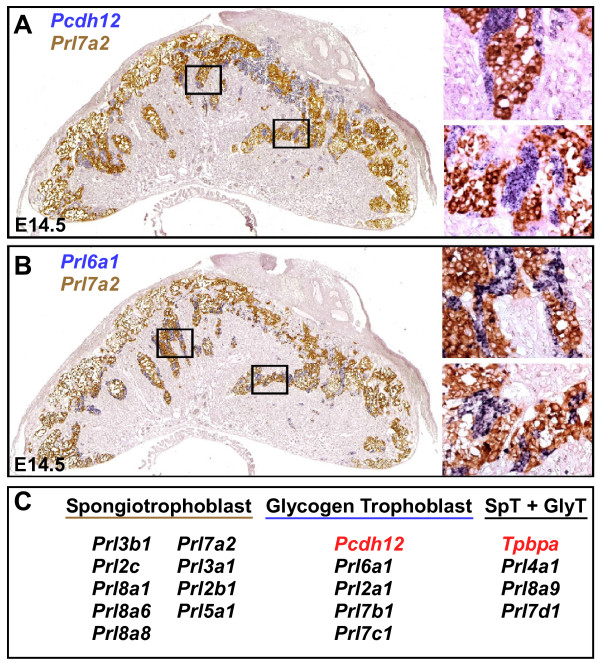
**Spongiotrophoblast and glycogen trophoblast expression of PRL/PL family members**. A – Double in situ hybridization of *Pcdh12 *(Blue) and *Prl7a2 *(Brown) in E14.5 placenta. B – Double in situ hybridization of *Prl6a1 *(Blue) and *Prl7a2 *(Brown) in E14.5 placenta. C – Summary of spongiotrophoblast and glycogen trophoblast-specific expression patterns as well as genes expressed in both populations.

We detected expression of several *PRL/PL*-related genes in the junctional zone of the placenta that contains spongiotrophoblast and glycogen trophoblast cells. Expression of the protocadherin gene, *Pcdh12*, has been shown to be specific to trophoblast glycogen cells in the murine placenta [44,45]. Therefore, *Pcdh12 *was used as a probe for double in situ hybridization analysis to assess whether *PRL/PL *family members expressed in the junctional zone were specific to spongiotrophoblast or to glycogen trophoblast cells (Fig 4). Surprisingly, most *PRL/PL*-related genes that were expressed within the junctional zone were either specifically expressed in spongiotrophoblast cells or in glycogen trophoblast cells (Fig 4), not in both populations as is the case for *Tpbpa *[46], a very widely used marker for these two cell types. *Prl3b1*, *Prl2c*, *Prl8a6*, *Prl8a8*, *Prl8a1*, *Prl7a2*, *Prl3a1*, *Prl2b1*, and *Prl5a1 *had complementary expression patterns to *Pcdh12*, indicating spongiotrophoblast cell specificity (Fig. 3). *Prl6a1*, *Prl2a1*, *Prl7b1 *and *Prl7c1 *all had expression patterns overlapping with *Pcdh12 *(or complementary to *Prl7a2 *or *Prl8a8 *as shown) indicating they were specifically expressed in glycogen trophoblast cells. The *Prl4a1*, *Prl8a9 *and *Prl7d1 *genes were expressed in both spongiotrophoblast and glycogen trophoblast cells (Fig. 4). Importantly, though, not all cells of a given subtype were positive for each gene. For example, only a small proportion of spongiotrophoblast and glycogen trophoblast cells were positive for *Prl4a1*, a gene more highly expressed in TGCs. *Prl6a1 *and *Prl7b1 *were both expressed in glycogen trophoblast cells specifically, but *Prl6a1 *was more prevalent in glycogen trophoblast cells that were resident within the junctional zone and less abundantly expressed in migrating glycogen cells located within the decidua. In contrast, *Prl7b1 *was more abundantly expressed in migratory glycogen trophoblast and spiral artery-associated TGCs throughout gestation.

### Common Regulatory Modules Within Unique Gene Promoter Regions Likely Convey Trophoblast Subtype-Specific Gene Expression

**Figure 5:**
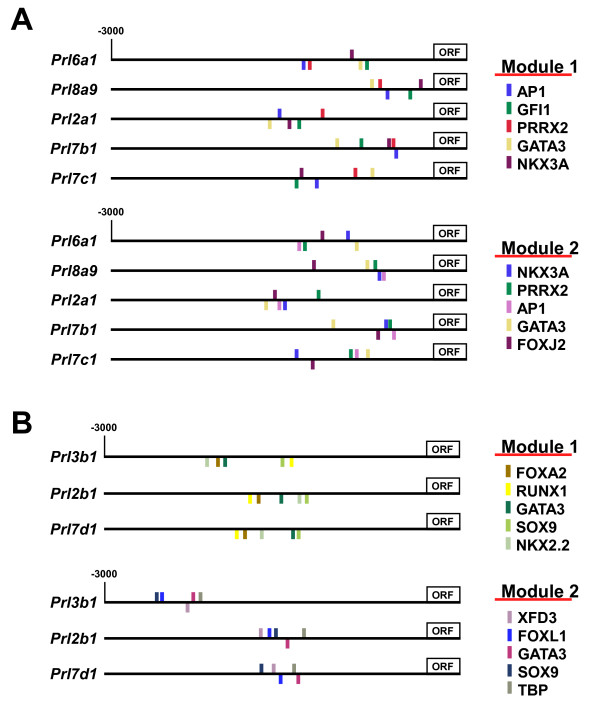
**Common modules of transcription factor binding sites within the promoters of commonly expressed PRL/PL family members**. A – 3000 bases upstream of the coding sequence plus 200 bases within the coding sequence of 5 genes that are expressed within the glycogen trophoblast population. B – 3000 bases upstream of the coding sequence plus 200 bases within the coding sequence of 3 genes that are expressed within the sinusoidal TGC population within the labyrinth. Examples of 2 different modules are shown.

A major aim of the current study was to gain insights into the transcriptional regulation of *PRL/PL*-related genes in the murine placenta. Despite a significantly expanded resolution of *PRL*/*PL *family member expression, our data set did not show any correlation between the expression patterns of genes and their proximity to one another within the locus, or between those most evolutionarily related. This is consistent with previously published studies [2,4] and bolsters the notion that local regulatory regions convey trophoblast subtype- and temporal-specific expression rather than locus control regions. We then used the Toucan2 bioinformatics program [37] to examine the upstream regulatory regions (~3000 nt) of genes co-expressed within trophoblast subtypes in an attempt to identify conserved groups, or modules, of putative transcription factor binding sites involved in subtype-specific expression. Genes robustly expressed in glycogen trophoblast cells (*Prl6a1*, *Prl2a1*, *Prl7b1*, *Prl7c1*, *Prl8a9*) were analyzed for modules of transcription factor binding sites that occur in all five promoter regions. Two modules were identified, each containing putative binding sites for five transcription factors within a region of ~600 base pairs (Fig. 5A). The two modules both contained sites for AP1, PRRX2, GATA3 and NKX3A. One of the modules also contained a GFI1 site whereas the other contained a FOXJ2 site. We also examined the promoters of three genes co-expressed in the sinusoidal TGC population (*Prl3b1*, *Prl2b1*, *Prl7d1*) and detected two conserved modules (Fig. 5B). Both modules contained sites for GATA3 and SOX9.

### Multiple Splice Variants for Some PRL/PL Family Members

The mouse genome sequence assemblies from the Ensembl [47] (m36-Jun2006) and Vega [48] (v19-Jun2006) databases predict the existence of splice variants for several PRL/PL family members, including *Prl6a1*, *Prl8a6*, *Prl8a8*, *Prl8a1 *and *Prl7a1*. In an effort to determine whether the predicted isoforms were expressed in vivo, sequences unique to each variant cDNA were used both to search EST databases and for primer design for RT-PCR analysis and cDNA sequencing.

#### Prl6a1

**Figure 6:**
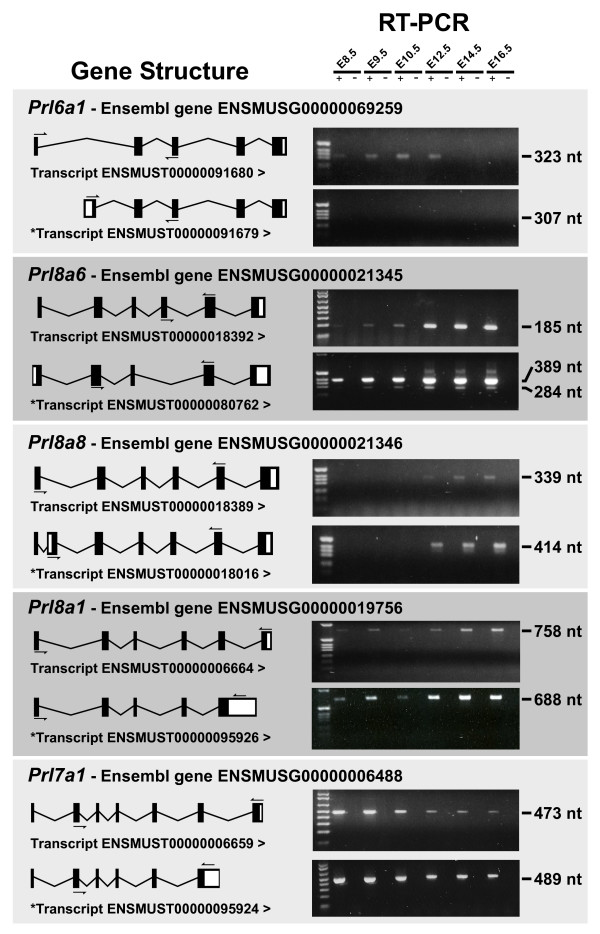
**Several PRL family members have multiple splice variants**. The splice patterns of both the originally reported transcripts and their predicted novel isoforms are depicted on the left. Arrows indicate the location and direction of PCR primers used to verify expression of each isoform. Arrow heads (>) indicate the direction of transcription. * indicates predicted transcript. Gel pictures showing PCR amplicons for all the isoforms with the corresponding fragment sizes are shown on the right.

Use of an alternative exon 1 was predicted both in the Ensembl assembly (transcript ENSMUST00000091679) and by the Vega database (named *Prl6a1*-002) (Fig. 6). Blast searches [49] using the predicted novel exon 1 sequence did not match any EST sequences in the NCBI or the institute for genomic research (TIGR) [50] databases. In addition, RT-PCR amplification using a forward primer specific to the alternative exon 1 and a common reverse primer recognizing exon 3 did not yield any bands, despite using cDNA generated from all stages of placental development and from three different genetic backgrounds (Fig. 6).

#### Prl8a6

The novel splice variant of *Prl8a6*, predicted by both the Ensembl (transcript ENSMUST00000080762) and Vega (*Prl8a6-001*) genome assemblies, has a unique 5' untranslated region (UTR) and an absence of exon 4 (Fig. 6). EST database searches using the predicted cDNA identified an EST from a placental cDNA library that was a perfect match (accession AK005478), suggesting that this isoform is expressed in vivo. RT-PCR analysis using primers flanking exon 4 produced two amplicons (Fig. 6) and sequencing of the PCR products revealed that the major upper band contained exon 4 and the lower band did not contain exon 4. Database searches revealed numerous ESTs that contained the novel 5' UTR sequence but were not missing exon 4. Since the entire published 5' UTR (transcript ENSMUST00000018392) is contained within predicted novel 5' UTR (ENSMUST00000080762), it is possible that the novel 5' UTR is not unique but reflects the incomplete 5' sequencing of ESTs in the databases corresponding to *Prl8a6*. However, RT-PCR analysis using a forward primer specific to the predicted 5' UTR of ENSMUST00000080762 produced a fragment which was expressed only at E14.5 and not at E9.5, suggesting that the novel 5' UTR sequence could be isoform specific (data not shown).

#### Prl8a8

Originally annotated and confirmed as a six exon gene, Ensembl and Vega assemblies both predict an additional isoform of *Prl8a8 *containing a novel exon between exons 1 and 2 (transcript ENSMUST00000018016) (Fig 6). NCBI database searches indicated 3 ESTs that contained the novel exon (accession CK022447, CK032606, CK020277) as well as over 33 ESTs that did not. The novel isoform had already been submitted to Genbank as *Prl8a8 isoform2 *(accession AF230923). RT-PCR analysis and sequencing of the PCR products confirmed expression of both isoforms in E14.5 placenta (Fig 6).

#### Prl8a1

An alternative isoform of *Prl8a1 *was predicted by both Ensembl (transcript ENSMUST00000095926) and Vega to terminate with an extended exon 5 rather than with exon 6, as is the case for the originally reported *Prl8a1 *(transcript ENSMUST00000006664) (Fig. 6). Searches of the NCBI and TIGR EST databases revealed an EST that was a 100% match with the predicted *Prl8a1 *novel isoform (accession AK014414). Our RT-PCR analysis indicated that both isoforms are expressed at E9.5 and E14.5, although similar to *Prl8a8*, the novel isoform is expressed at a lower level (Fig. 6).

#### Prl7a1

An alternative isoform of *Prl7a1 *was predicted by the Ensembl assembly (transcript ENSMUST00000095924) to terminate with an extended exon 6 rather than with exon 7, as is the case for the originally reported *Prl7a1 *(transcript ENSMUST00000006659) (Fig. 6). An EST search returned a match with the novel isoform (accession AK028371). In addition, RT-PCR using primers specific to either the extended exon 6 or to exon 7, coupled with a common forward primer for exon2, could amplify transcripts from both isoforms (confirmed by sequencing) at E9.5 and E14.5 (Fig. 6).

### Multiple Prl2c Genes Exist, Located Both Inside and Outside the PRL/PL Family Locus

Between four and six *Prl2c *genes are predicted to exist within the mouse genome [51,52]. Four unique cDNAs have been described [40,41,52], which correspond to the four *Prl2c *genes currently annotated in the mouse genome: *Prl2c2*, *Prl2c3*, *Prl2c4 *and *Prl2c5*. Notably, all of these four genes are located outside of the main *PRL/PL *locus but are still on chromosome 13. Three of the genes are located approximately 10 Mb away from the main *PRL/PL *locus; *Prl2c4 *is found at 12.88 Mb, *Prl2c2 *(*MRP-1*/*Plf-1*) is found at 13.09 Mb and *Prl2c5 *(Formerly *Mrpplf4*, *MRP-4 *and *Plf-4*) at 13.27 Mb (Fig 2), representing a *Prl2c *cluster outside the boundaries of the main *PRL/PL *locus. Some discrepancies and confusion remain regarding the *Prl2c *genes, however, as searches of Ensembl show that *Prl2c3 *is listed as an alternative name for *Prl2c4 *(also formerly *Mrpplf3*, *MRP-2*, *Plf-2 *and *Plf-3*). Originally, detailed mapping of the mouse *PRL/PL *locus [2,4] had identified only a single *Prl2c *gene located between *Prl2a1 *and *Prl4a1 *within the PRL/PL cluster on chromosome 13. Only a single gene was identified in the rat PRL/PL cluster also [5]. Indeed, a candidate *Prl2c *gene exists in this location in mice (ENSMUSG00000062551) and is currently predicted to have several alternative splice variants. Interestingly, to our knowledge, cDNAs corresponding to these predicted transcripts have yet to be described. Overall, it is very likely that the nomenclature will have to be revised as the existence of additional *Prl2c *genes located inside and/or outside of the *PRL/PL *locus is verified.

## Discussion

In the present study, a detailed temporal and spatial placental expression map was generated for all murine *PRL/PL *family members from E7.5 to E18.5 of gestation in three genetic strains. This detailed analysis uncovered several new markers for some trophoblast cell types that will be useful for future analysis of placental structure in mutant mice with placental phenotypes. Moreover, several important conclusions about regulation of the locus are apparent. First, no two family members have the same expression pattern even when complete temporal and spatial data are examined. Second, most genes are expressed in multiple trophoblast cell subtypes though none were detected in the chorion, where trophoblast stem cells reside, or in syncytiotrophoblast of the labyrinth layer. Third, bioinformatic comparisons of upstream regulatory regions identified predicted transcription factor binding site modules that are shared by genes expressed in the same trophoblast subtype. Fourth, further diversification of gene products from the *PRL/PL *locus occurs through alternative splice isoforms for several genes.

In reviewing the summary data, it was striking that so many genes within the *PRL/PL *family are expressed in the ectoplacental cone and in the adjacent parietal TGCs early in gestation. It was already reported that TGCs at the periphery of the ectoplacental cone express *Prl3d *and *Prl2c *as early as E6.5 [53] and *Prl7a1*, *Prl4a1 *and *Prl6a1 *are expressed in the developing placenta as early as E8.0 [32,54]. Interestingly, *PRL/PL *family members found at the periphery of the ectoplacental cone are not uniformly expressed in all TGCs and likely demarcate distinct TGC subpopulations and/or stages of TGC differentiation. Expression of genes in trophoblast cells at the center of the ectoplacental cone was also diverse. Of note, *Prl3b1 *is expressed in a small subset of cells within the center of the ectoplacental cone at E8.5, possibly in secondary TGC precursors or spongiotrophoblast precursors. *Prl3b1 *expression, which is broad later in gestation, has not been described before E9 at which time *Prl3d *expression in TGCs begins to decline [53]. *Prl5a1 *expression is also interesting as it is biphasic in its expression, expressed in both TGCs and cells in the centre of the ectoplacental cone early in gestation and not again until late in gestation where it is expressed in spongiotrophoblast cells. Further studies are required to understand the heterogeneous ectoplacental cone and parietal TGC cell populations.

The current studies have also provided several markers that are restricted to either the spongiotrophoblast or glycogen cell populations. The junctional zone of the placenta contains both spongiotrophoblast cells and glycogen trophoblast cells. While many genes are known to be expressed within this layer [35], there had been very few genes definitively characterized as markers of either spongiotrophoblast or glycogen trophoblast cells, although members of the *PRL/PL *family (*Prl2a1*, *Prl7b1 *in mouse and *Prl4a1*, *Prl2a1*, *Prl7b1 *and *Prl5a1 *in rat) have been specifically localized to invasive/migratory trophoblast within the decidua [55], suggesting 'migratory' or glycogen trophoblast specificity. *Pcdh12 *[44,45] and connexin 31 [56] have been recently identified as glycogen trophoblast cell-specific markers, but no spongiotrophoblast-specific markers have been reported. Double in situ hybridization with *PRL/PL *family members and *Pcdh12 *showed that expression of *Prl3b1*, *Prl2c*, *Prl8a6*, *Prl8a8*, *Prl8a1*, *Prl7a2*, *Prl3a1*, *Prl2b1*, and *Prl5a1 *is restricted to spongiotrophoblast cells. *Prl8a8 *appears to be the best spongiotrophoblast-specific marker as it is not expressed in any other trophoblast cell subtypes throughout gestation and appears to be broadly expressed within the entire population of spongiotrophoblast. In contrast, *Prl6a1*, *Prl2a1*, *Prl7b1 *and *Prl7c1 *all have expression patterns overlapping with *Pcdh12 *expression (or complementary to *Prl7a2 *as shown) indicating they are specifically expressed in glycogen trophoblast cells within this layer. Markers that allow the distinction of glycogen trophoblast and spongiotrophoblast cells will be very useful in dissecting the pathology of mouse mutants with placental phenotypes, since the most commonly used marker, *Tpbpa*, is expressed in both [46]. It is also unclear whether glycogen trophoblast cells differentiate directly from spongiotrophoblast later in gestation [46] or whether they represent an independent trophoblast population earlier within the ectoplacental cone [44]. Examining both spongiotrophoblast and glycogen trophoblast in mutant mice with defects in the junctional zone will give insights into this question.

Gaining a better understanding of the regulation of gene expression from the PRL/PL locus was also a major aim of our study. Placental-specific expression from the expanded human growth hormone locus is regulated by a locus control region 15–32 kb upstream of the gene cluster [26,27]. The evidence to date has not generally supported the notion that the rodent *PRL*/*PL *genes are similarly regulated; small defined upstream regions isolated from the promoters of several *PRL*/*PL *family members, including rat *Prl3b1 *(*Pl2*) [57-59], mouse *Prl3d *(*Pl1*) [60-62] and *Prl2c *(*Plf*) [60], rat *Prl4a1 *(*Plp-A*) [63], rat *Prl8a2 *(*d/tPrp*) [64], rat *Prl8a3 *(*Plp-Cv*) [65], independently drive trophoblast-specific expression in vitro. Furthermore, genes with similar placental expression patterns do not appear to be grouped together within the *PRL*/*PL *cluster [2,4]. Nevertheless, regulatory elements for *Prl3b1 *[66] and *Pl3d *and *Prl2c *(J.C. Cross, unpublished data) that are sufficient in vitro fail to drive trophoblast-specific expression when tested in vivo. This indicates a requirement for additional elements for these genes at least making it difficult to rule out completely the involvement of locus control regions or additional enhancers. In addition, complete temporal and spatial expression data for each *PRL*/*PL *family member have not been compiled. In the current study we compiled expression data for each family member over the whole time course of placentation with high resolution, reporting expression patterns in all the characterized trophoblast subtypes, in an effort to uncover unappreciated associations between locus position and expression patterns. The identification of regulatory elements driving trophoblast subtype-specific gene expression would be of particular use for future studies of trophoblast differentiation and placental development. It is now clear from our data set however that each *PRL*/*PL *family member has a truly unique expression pattern with no correlation to locus structure, consistent with previous reports [2,4] and diminishing further the likelihood of control regions driving subtype-specific trophoblast expression. As such, subtype-specific expression patterns are likely driven by elements contained within the local upstream promoters of individual genes, although this does not preclude the possibility of a locus control region regulating placental-specific for the entire cluster, regulating trophoblast-specific transcriptional access to the whole of the region.

We therefore sought another way to investigate trophoblast subtype-specific regulatory elements. Previous studies of *PRL*/*PL *gene regulation have rarely investigated what, if any, subtype-specific expression is conveyed by the identified elements. Two notable exceptions are an enhancer element from the rat *Prl8a3 *promoter shown to drive expression preferentially in spongiotrophoblast cells rather than TGCs [65] and the demonstration that the transcription factor Gata2 is essential to restrict *Prl4a1 *expression to secondary TGCs within the TGC population [67]. We used the bioinformatics software program Toucan2 [37] to identify groups, or modules, of putative transcription factor binding sites present in multiple promoter sequences from genes co-expressed in a particular trophoblast subtype, rather than a single promoter in isolation. This approach effectively narrows the focus to elements involved in gene expression within a particular cell population, rather than trophoblast specificity in general. Our comprehensive expression data set allowed us for the first time to try such an in silico approach and we compared the promoters of genes co-expressed in the poorly characterized glycogen trophoblast and sinusoidal TGC populations as examples, identifying several modules of putative transcription factor binding sites common to the promoters of each group. The identification of these conserved modules serves not only to bolster the notion of local versus distant regulation of *PRL*/*PL *family members, but provides a valuable in silico resource to identify candidate elements for further experimental studies. Interestingly, putative Gata3 sites were identified in all the modules, a factor of known importance for trophoblast-specific expression of *Prl3d *and *Prl2c *[60-62]. Also, putative AP-1 sites were present in both modules contained in the promoters of genes co-expressed in glycogen trophoblast cells, motifs important for *Plr3d *and *Plr3b1 *expression [59,62]. The presence of motifs previously been shown to play roles in regulating trophoblast-specific *PRL*/PL members within the modules imparts a higher degree of confidence to these predicted elements, making them strong candidates for further experimental studies.

We conducted our expression analysis in three different mouse lines, not only to increase the confidence of our data set but also to investigate whether any *PRL/PL *family members are expressed differently between commonly used strains. We chose the outbred stock CD-1 and two inbred strains C57/B6 and 129svj, all commonly used lines. Average litter sizes are known to vary among different mouse stocks and strains (129svj – average 4.5 pups/litter [68], C57/B6 – average 6.2 pups/litter [68], CD-1 – average 11 pups/litter (Charles River) and several PRL/PL family members have been shown to regulate reproductive adaptations to physiological stresses such as hypoxia. Differences in PRL/PL gene expression between lines may therefore be related to differences in litter size and the accompanying changes in physiological adaptation [69]. Interestingly, only a few slight differences in the timing of *PRL*/*PL *family expression or the breadth of expression within a certain trophoblast subtype were observed.

Gene duplication followed by the acquisition of novel gene function offers a way for species to adapt to changing environmental challenges or to capitalize on newly available niches. Interestingly, members of the murine *PRL/PL *family have undergone further diversification by adopting splice variants. Evidence for positive selection within large families of amplified genes, particularly those associated with reproduction, has been accumulating [70-74]. One theory suggests that the gene duplication event itself is positively selected for to allow the amplification of genes that are somewhat pre-adapted to meet a particular environmental challenge or biochemical niche, so that divergence and acquisition of new functionality may follow [75]. It is tempting to visualize the evolution of the *PRL/PL *genes in rodents and ruminants as a result of the driving environmental challenge of reproductive fitness. Numerous other gene families have been reported in the mouse genome that are also associated with reproduction such as the placentally expressed cathepsins [76-78], Rhox transcription factors [79] and pregnancy-specific glycoproteins [80,81], although their specific roles in reproduction remain largely unknown. The functions of the individual *PRL/PL *family members are just beginning to be revealed through knockout mouse studies. The diverse patterns of expression and evidence of splice variants indicates that the family is rapidly evolving and suggests that the different genes have distinct functions. The majority of *PRL/PL *genes are expressed later in gestation when it is likely they are acting as hormones to affect feto-maternal adaptations to pregnancy, but it is also apparent from this study that approximately half of the *PRL/PL *family members are expressed early in the ectoplacental cone and may be involved in decisions affecting cell fate. Whether the *PRL/PL *locus evolved as a result of positive selection, or some other adaptive force, it is now clear that PRL/PL gene amplification serves as an excellent model for studying the process of genetic diversification.

## Conclusion

The gene expression data in this study clearly demonstrates that no two PRL/PL family members share the same temporal and spatial expression pattern. It is now very clear that there is no correlation in expression between genes that are most closely related or between adjacent genes in the PRL/PL locus. Bioinformatic analysis of upstream regulatory regions identified conserved modules of putative transcription factor binding sites shared by genes expressed in the same trophoblast subtype. Interestingly, we have also observed that although mouse PRL/PL genes are expressed in multiple trophoblast subtypes, no family members were detected in the chorion, where trophoblast stem cells reside, or in syncytiotrophoblast of the labyrinth layer. The information gleaned in this study has also identified novel markers that will provide valuable insight into mouse models with placental phenotypes. Additionally, we have observed for the first time that approximately half of the PRL/PL genes are expressed early in the ectoplacental cone and may be involved in decisions affecting cell fate. Based on our findings, we propose that the PRL/PL gene family represents an excellent model for studying the process of genetic diversification.

## Authors' contributions

DGS carried out the majority of the molecular genetic studies and drafted the manuscript. SR assisted with in situ hybridizations, conceived of and assisted in the alternative isoform study, performed bioinformatics analysis and participated in drafting the manuscript. AD assisted in data analysis and collation. MH performed northern blot analysis. JCC participated in the design and coordination of this study and helped to draft the manuscript.

## Supplementary Material

Additional file 1 **Trophoblast subtype-specific expression profiles of PRL family members compared with location within the PRL family locus**. Blue boxes indicate positive expression of the corresponding gene within the designated trophoblast subtype population, although there is no indication of the proportion of cells within a given population that are positive. Grey boxes indicate no gene expression within a given trophoblast subtype. Genes labeled in red indicate those genes which are expressed in only one trophoblast subtype while black labels indicate genes expressed in multiple subtypes. Rectangles are used to indicate the location of genes within the locus on chromosome 13 while ovals are used to indicate the location of pseudogenes. *The *Prl2c *gene located within the main prolactin family cluster (27–28 Mb) does not correspond to any of the 4 *Prl2c *genes previously annotated from cDNA sequences (*Prl2c2, c3, c4 and c5*) and is therefore referred to as *Plf*, the original gene symbol. Summary of spatial expression data for each *PRL*/*PL *family member correlated with gene position within the *PRL*/*PL *locus. Click here for file

Additional file 2 A – In situ hybridizations of early (E8.5) and mid to late gestation (E12.5, E14.5, or E18.5) placenta for each member of the PRL/PL family. Higher magnifications emphasize particular trophoblast subtypes including parietal TGCs, spiral artery TGCs, canal TGCs, sinusoidal TGCs, spongiotrophoblast, glycogen trophoblast cells, and decidua. B – Temporal gene expression data (based in situ hybridization signals) for individual placental cell types. Shades of grey depict an estimation of the percentage of each cell type that expresses the gene. White – 0%, Light grey ~25%, Medium Grey ~50%, Dark grey ~75%, Black > 75%. Summary of in situ hybridization data for the *Prl3d *genes. Click here for file

Additional file 3 A – In situ hybridizations of early (E8.5) and mid to late gestation (E12.5, E14.5, or E18.5) placenta for each member of the PRL/PL family. Higher magnifications emphasize particular trophoblast subtypes including parietal TGCs, spiral artery TGCs, canal TGCs, sinusoidal TGCs, spongiotrophoblast, glycogen trophoblast cells, and decidua. B – Temporal gene expression data (based in situ hybridization signals) for individual placental cell types. Shades of grey depict an estimation of the percentage of each cell type that expresses the gene. White – 0%, Light grey ~25%, Medium Grey ~50%, Dark grey ~75%, Black > 75%. Summary of in situ hybridization data for *Prl3c1*. Click here for file

Additional file 4 A – In situ hybridizations of early (E8.5) and mid to late gestation (E12.5, E14.5, or E18.5) placenta for each member of the PRL/PL family. Higher magnifications emphasize particular trophoblast subtypes including parietal TGCs, spiral artery TGCs, canal TGCs, sinusoidal TGCs, spongiotrophoblast, glycogen trophoblast cells, and decidua. B – Temporal gene expression data (based in situ hybridization signals) for individual placental cell types. Shades of grey depict an estimation of the percentage of each cell type that expresses the gene. White – 0%, Light grey ~25%, Medium Grey ~50%, Dark grey ~75%, Black > 75%. Summary of in situ hybridization data for *Prl3b1*. Click here for file

Additional file 5 A – In situ hybridizations of early (E8.5) and mid to late gestation (E12.5, E14.5, or E18.5) placenta for each member of the PRL/PL family. Higher magnifications emphasize particular trophoblast subtypes including parietal TGCs, spiral artery TGCs, canal TGCs, sinusoidal TGCs, spongiotrophoblast, glycogen trophoblast cells, and decidua. B – Temporal gene expression data (based in situ hybridization signals) for individual placental cell types. Shades of grey depict an estimation of the percentage of each cell type that expresses the gene. White – 0%, Light grey ~25%, Medium Grey ~50%, Dark grey ~75%, Black > 75%. Summary of in situ hybridization data for *Prl3a1*. Click here for file

Additional file 6 A – In situ hybridizations of early (E8.5) and mid to late gestation (E12.5, E14.5, or E18.5) placenta for each member of the PRL/PL family. Higher magnifications emphasize particular trophoblast subtypes including parietal TGCs, spiral artery TGCs, canal TGCs, sinusoidal TGCs, spongiotrophoblast, glycogen trophoblast cells, and decidua. B – Temporal gene expression data (based in situ hybridization signals) for individual placental cell types. Shades of grey depict an estimation of the percentage of each cell type that expresses the gene. White – 0%, Light grey ~25%, Medium Grey ~50%, Dark grey ~75%, Black > 75%. Summary of in situ hybridization data for *Prl6a1*. Click here for file

Additional file 7 A – In situ hybridizations of early (E8.5) and mid to late gestation (E12.5, E14.5, or E18.5) placenta for each member of the PRL/PL family. Higher magnifications emphasize particular trophoblast subtypes including parietal TGCs, spiral artery TGCs, canal TGCs, sinusoidal TGCs, spongiotrophoblast, glycogen trophoblast cells, and decidua. B – Temporal gene expression data (based in situ hybridization signals) for individual placental cell types. Shades of grey depict an estimation of the percentage of each cell type that expresses the gene. White – 0%, Light grey ~25%, Medium Grey ~50%, Dark grey ~75%, Black > 75%. Summary of in situ hybridization data for *Prl8a2*. Click here for file

Additional file 8 A – In situ hybridizations of early (E8.5) and mid to late gestation (E12.5, E14.5, or E18.5) placenta for each member of the PRL/PL family. Higher magnifications emphasize particular trophoblast subtypes including parietal TGCs, spiral artery TGCs, canal TGCs, sinusoidal TGCs, spongiotrophoblast, glycogen trophoblast cells, and decidua. B – Temporal gene expression data (based in situ hybridization signals) for individual placental cell types. Shades of grey depict an estimation of the percentage of each cell type that expresses the gene. White – 0%, Light grey ~25%, Medium Grey ~50%, Dark grey ~75%, Black > 75%. Summary of in situ hybridization data for *Prl2b1*. Click here for file

Additional file 9 A – In situ hybridizations of early (E8.5) and mid to late gestation (E12.5, E14.5, or E18.5) placenta for each member of the PRL/PL family. Higher magnifications emphasize particular trophoblast subtypes including parietal TGCs, spiral artery TGCs, canal TGCs, sinusoidal TGCs, spongiotrophoblast, glycogen trophoblast cells, and decidua. B – Temporal gene expression data (based in situ hybridization signals) for individual placental cell types. Shades of grey depict an estimation of the percentage of each cell type that expresses the gene. White – 0%, Light grey ~25%, Medium Grey ~50%, Dark grey ~75%, Black > 75%. Summary of in situ hybridization data for *Prl8a6*. Click here for file

Additional file 10 A – In situ hybridizations of early (E8.5) and mid to late gestation (E12.5, E14.5, or E18.5) placenta for each member of the PRL/PL family. Higher magnifications emphasize particular trophoblast subtypes including parietal TGCs, spiral artery TGCs, canal TGCs, sinusoidal TGCs, spongiotrophoblast, glycogen trophoblast cells, and decidua. B – Temporal gene expression data (based in situ hybridization signals) for individual placental cell types. Shades of grey depict an estimation of the percentage of each cell type that expresses the gene. White – 0%, Light grey ~25%, Medium Grey ~50%, Dark grey ~75%, Black > 75%. Summary of in situ hybridization data for *Prl8a8*. Click here for file

Additional file 11 A – In situ hybridizations of early (E8.5) and mid to late gestation (E12.5, E14.5, or E18.5) placenta for each member of the PRL/PL family. Higher magnifications emphasize particular trophoblast subtypes including parietal TGCs, spiral artery TGCs, canal TGCs, sinusoidal TGCs, spongiotrophoblast, glycogen trophoblast cells, and decidua. B – Temporal gene expression data (based in situ hybridization signals) for individual placental cell types. Shades of grey depict an estimation of the percentage of each cell type that expresses the gene. White – 0%, Light grey ~25%, Medium Grey ~50%, Dark grey ~75%, Black > 75%. Summary of in situ hybridization data for *Prl8a9*. Click here for file

Additional file 12 A – In situ hybridizations of early (E8.5) and mid to late gestation (E12.5, E14.5, or E18.5) placenta for each member of the PRL/PL family. Higher magnifications emphasize particular trophoblast subtypes including parietal TGCs, spiral artery TGCs, canal TGCs, sinusoidal TGCs, spongiotrophoblast, glycogen trophoblast cells, and decidua. B – Temporal gene expression data (based in situ hybridization signals) for individual placental cell types. Shades of grey depict an estimation of the percentage of each cell type that expresses the gene. White – 0%, Light grey ~25%, Medium Grey ~50%, Dark grey ~75%, Black > 75%. Summary of in situ hybridization data for *Prl8a1*. Click here for file

Additional file 13 A – In situ hybridizations of early (E8.5) and mid to late gestation (E12.5, E14.5, or E18.5) placenta for each member of the PRL/PL family. Higher magnifications emphasize particular trophoblast subtypes including parietal TGCs, spiral artery TGCs, canal TGCs, sinusoidal TGCs, spongiotrophoblast, glycogen trophoblast cells, and decidua. B – Temporal gene expression data (based in situ hybridization signals) for individual placental cell types. Shades of grey depict an estimation of the percentage of each cell type that expresses the gene. White – 0%, Light grey ~25%, Medium Grey ~50%, Dark grey ~75%, Black > 75%. Summary of in situ hybridization data for *Prl7b1*. Click here for file

Additional file 14 A – In situ hybridizations of early (E8.5) and mid to late gestation (E12.5, E14.5, or E18.5) placenta for each member of the PRL/PL family. Higher magnifications emphasize particular trophoblast subtypes including parietal TGCs, spiral artery TGCs, canal TGCs, sinusoidal TGCs, spongiotrophoblast, glycogen trophoblast cells, and decidua. B – Temporal gene expression data (based in situ hybridization signals) for individual placental cell types. Shades of grey depict an estimation of the percentage of each cell type that expresses the gene. White – 0%, Light grey ~25%, Medium Grey ~50%, Dark grey ~75%, Black > 75%. Summary of in situ hybridization data for *Prl7a1*. Click here for file

Additional file 15 A – In situ hybridizations of early (E8.5) and mid to late gestation (E12.5, E14.5, or E18.5) placenta for each member of the PRL/PL family. Higher magnifications emphasize particular trophoblast subtypes including parietal TGCs, spiral artery TGCs, canal TGCs, sinusoidal TGCs, spongiotrophoblast, glycogen trophoblast cells, and decidua. B – Temporal gene expression data (based in situ hybridization signals) for individual placental cell types. Shades of grey depict an estimation of the percentage of each cell type that expresses the gene. White – 0%, Light grey ~25%, Medium Grey ~50%, Dark grey ~75%, Black > 75%. Summary of in situ hybridization data for *Prl7a2*. Click here for file

Additional file 16 A – In situ hybridizations of early (E8.5) and mid to late gestation (E12.5, E14.5, or E18.5) placenta for each member of the PRL/PL family. Higher magnifications emphasize particular trophoblast subtypes including parietal TGCs, spiral artery TGCs, canal TGCs, sinusoidal TGCs, spongiotrophoblast, glycogen trophoblast cells, and decidua. B – Temporal gene expression data (based in situ hybridization signals) for individual placental cell types. Shades of grey depict an estimation of the percentage of each cell type that expresses the gene. White – 0%, Light grey ~25%, Medium Grey ~50%, Dark grey ~75%, Black > 75%. Summary of in situ hybridization data for *Prl7d1*. Click here for file

Additional file 17 A – In situ hybridizations of early (E8.5) and mid to late gestation (E12.5, E14.5, or E18.5) placenta for each member of the PRL/PL family. Higher magnifications emphasize particular trophoblast subtypes including parietal TGCs, spiral artery TGCs, canal TGCs, sinusoidal TGCs, spongiotrophoblast, glycogen trophoblast cells, and decidua. B – Temporal gene expression data (based in situ hybridization signals) for individual placental cell types. Shades of grey depict an estimation of the percentage of each cell type that expresses the gene. White – 0%, Light grey ~25%, Medium Grey ~50%, Dark grey ~75%, Black > 75%. Summary of in situ hybridization data for *Prl7c1*. Click here for file

Additional file 18 A – In situ hybridizations of early (E8.5) and mid to late gestation (E12.5, E14.5, or E18.5) placenta for each member of the PRL/PL family. Higher magnifications emphasize particular trophoblast subtypes including parietal TGCs, spiral artery TGCs, canal TGCs, sinusoidal TGCs, spongiotrophoblast, glycogen trophoblast cells, and decidua. B – Temporal gene expression data (based in situ hybridization signals) for individual placental cell types. Shades of grey depict an estimation of the percentage of each cell type that expresses the gene. White – 0%, Light grey ~25%, Medium Grey ~50%, Dark grey ~75%, Black > 75%. Summary of in situ hybridization data for *Prl2a1*. Click here for file

Additional file 19 A – In situ hybridizations of early (E8.5) and mid to late gestation (E12.5, E14.5, or E18.5) placenta for each member of the PRL/PL family. Higher magnifications emphasize particular trophoblast subtypes including parietal TGCs, spiral artery TGCs, canal TGCs, sinusoidal TGCs, spongiotrophoblast, glycogen trophoblast cells, and decidua. B – Temporal gene expression data (based in situ hybridization signals) for individual placental cell types. Shades of grey depict an estimation of the percentage of each cell type that expresses the gene. White – 0%, Light grey ~25%, Medium Grey ~50%, Dark grey ~75%, Black > 75%. Summary of in situ hybridization data for the *Prl2c *genes. Click here for file

Additional file 20 A – In situ hybridizations of early (E8.5) and mid to late gestation (E12.5, E14.5, or E18.5) placenta for each member of the PRL/PL family. Higher magnifications emphasize particular trophoblast subtypes including parietal TGCs, spiral artery TGCs, canal TGCs, sinusoidal TGCs, spongiotrophoblast, glycogen trophoblast cells, and decidua. B – Temporal gene expression data (based in situ hybridization signals) for individual placental cell types. Shades of grey depict an estimation of the percentage of each cell type that expresses the gene. White – 0%, Light grey ~25%, Medium Grey ~50%, Dark grey ~75%, Black > 75%. Summary of in situ hybridization data for *Prl4a1*. Click here for file

Additional file 21 A – In situ hybridizations of early (E8.5) and mid to late gestation (E12.5, E14.5, or E18.5) placenta for each member of the PRL/PL family. Higher magnifications emphasize particular trophoblast subtypes including parietal TGCs, spiral artery TGCs, canal TGCs, sinusoidal TGCs, spongiotrophoblast, glycogen trophoblast cells, and decidua. B – Temporal gene expression data (based in situ hybridization signals) for individual placental cell types. Shades of grey depict an estimation of the percentage of each cell type that expresses the gene. White – 0%, Light grey ~25%, Medium Grey ~50%, Dark grey ~75%, Black > 75%. Summary of in situ hybridization data for *Prl5a1*. Click here for file
