# Supplementary figures and images for: Spatial and temporal expression of the 23 murine Prolactin/Placental Lactogen-related genes is not associated with their position in the locus
Source: BMC Genomics. 2008 Jul 28;9:352. doi: 10.1186/1471-2164-9-352 (PMC2527339; doi:10.1186/1471-2164-9-352)

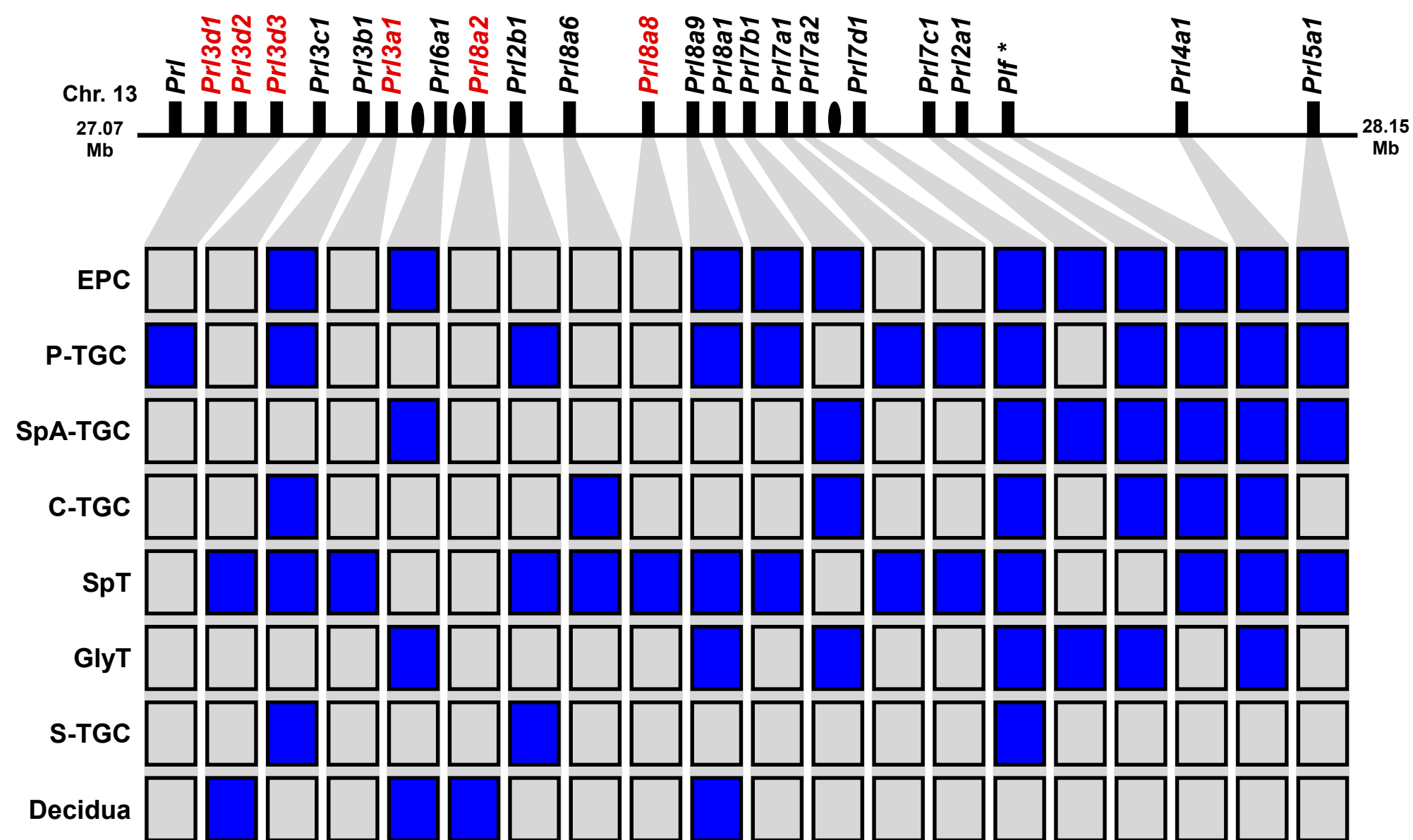

Supplement: Additional file 1 — Trophoblast subtype-specific expression profiles of PRL family members compared with location within the PRL family locus. Blue boxes indicate positive expression of the corresponding gene within the designated trophoblast subtype population, although there is no indication of the proportion of cells within a given population that are positive. Grey boxes indicate no gene expression within a given trophoblast subtype. Genes labeled in red indicate those genes which are expressed in only one trophoblast subtype while black labels indicate genes expressed in multiple subtypes. Rectangles are used to indicate the location of genes within the locus on chromosome 13 while ovals are used to indicate the location of pseudogenes. *The Prl2c gene located within the main prolactin family cluster (27–28 Mb) does not correspond to any of the 4 Prl2c genes previously annotated from cDNA sequences (Prl2c2, c3, c4 and c5) and is therefore referred to as Plf, the original gene symbol. Summary of spatial expression data for each PRL/PL family member correlated with gene position within the PRL/PL locus. [file 1471-2164-9-352-S1.pdf]

Gene: *Prl3d1* (also *Prl3d2* and *Prl3d3*) (Formally *Pl1*)

A

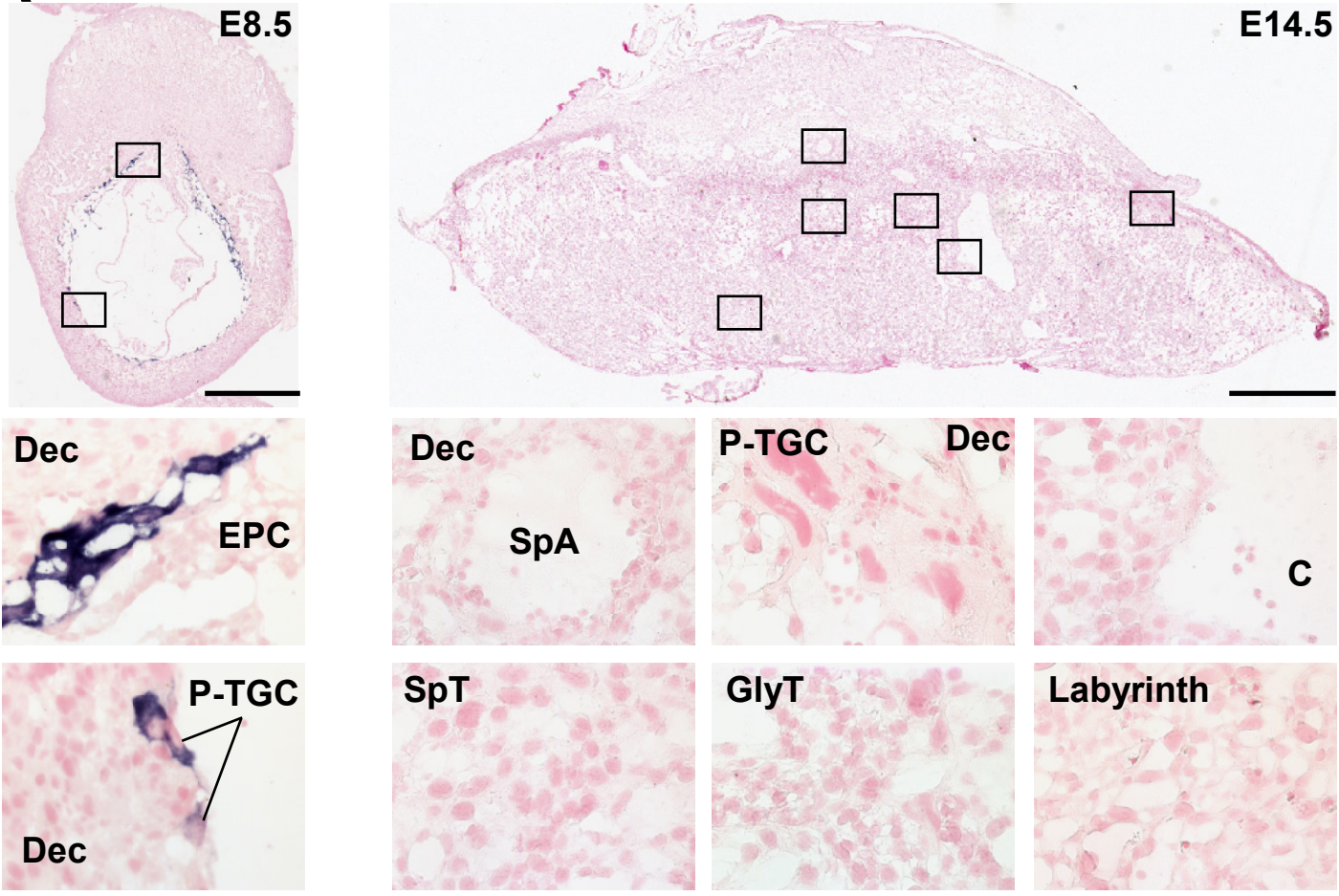

B

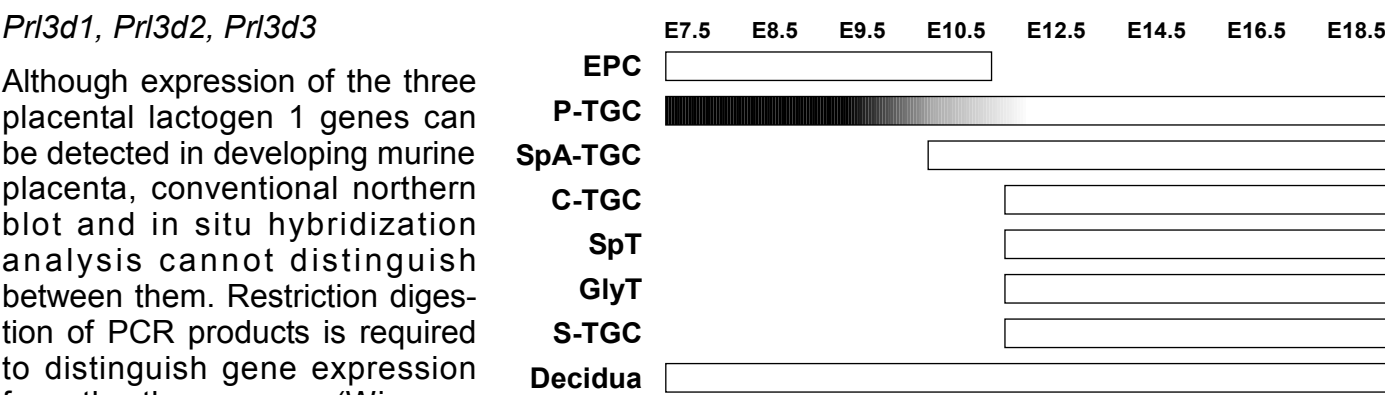

Supplement: Additional file 2 — A – In situ hybridizations of early (E8.5) and mid to late gestation (E12.5, E14.5, or E18.5) placenta for each member of the PRL/PL family. Higher magnifications emphasize particular trophoblast subtypes including parietal TGCs, spiral artery TGCs, canal TGCs, sinusoidal TGCs, spongiotrophoblast, glycogen trophoblast cells, and decidua. B – Temporal gene expression data (based in situ hybridization signals) for individual placental cell types. Shades of grey depict an estimation of the percentage of each cell type that expresses the gene. White – 0%, Light grey ~25%, Medium Grey ~50%, Dark grey ~75%, Black > 75%. Summary of in situ hybridization data for the Prl3d genes. [file 1471-2164-9-352-S2.pdf]

Gene: *Prl3c1* (*Prlpj*)

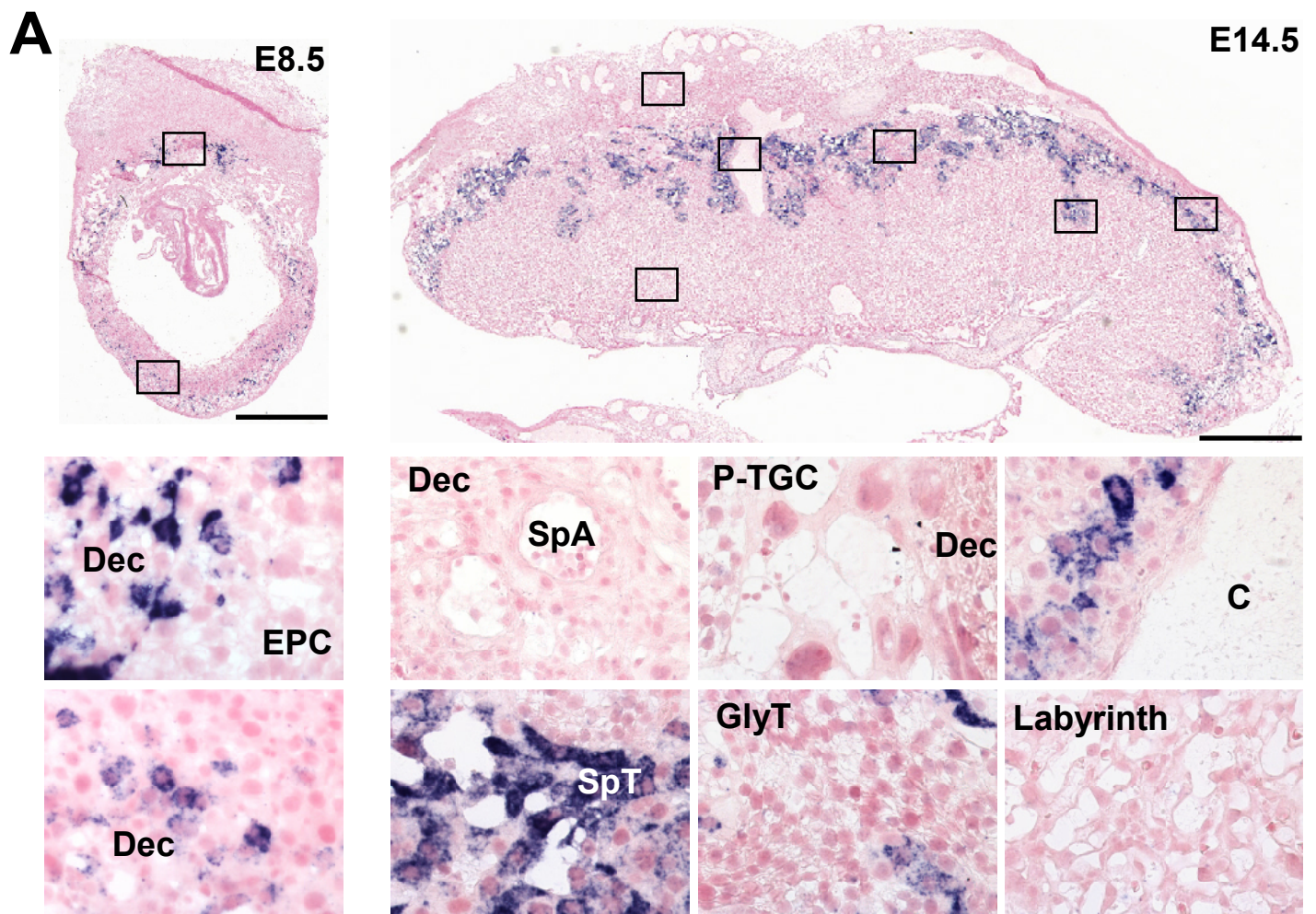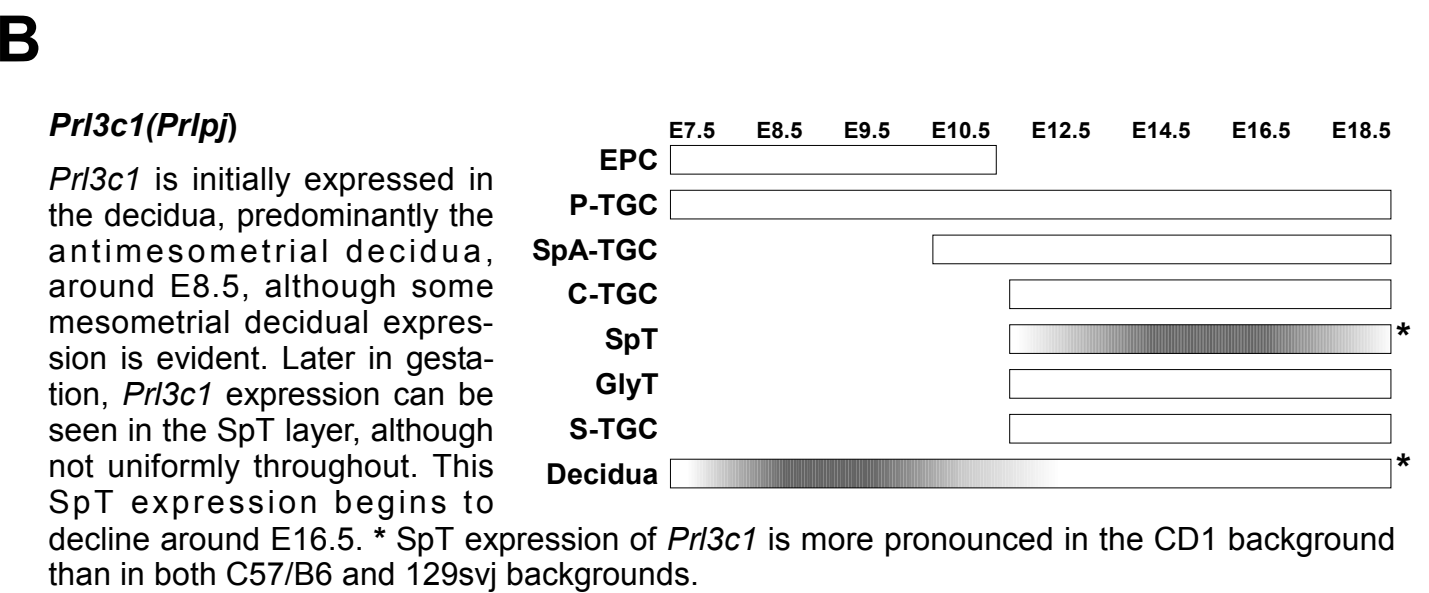

Previous publications showing mouse *Prl3c1* expression: (Dai et al., 2000)

Supplement: Additional file 3 — A – In situ hybridizations of early (E8.5) and mid to late gestation (E12.5, E14.5, or E18.5) placenta for each member of the PRL/PL family. Higher magnifications emphasize particular trophoblast subtypes including parietal TGCs, spiral artery TGCs, canal TGCs, sinusoidal TGCs, spongiotrophoblast, glycogen trophoblast cells, and decidua. B – Temporal gene expression data (based in situ hybridization signals) for individual placental cell types. Shades of grey depict an estimation of the percentage of each cell type that expresses the gene. White – 0%, Light grey ~25%, Medium Grey ~50%, Dark grey ~75%, Black > 75%. Summary of in situ hybridization data for Prl3c1. [file 1471-2164-9-352-S3.pdf]

# Gene: *Prl7b1* (*Prlpn*)

A

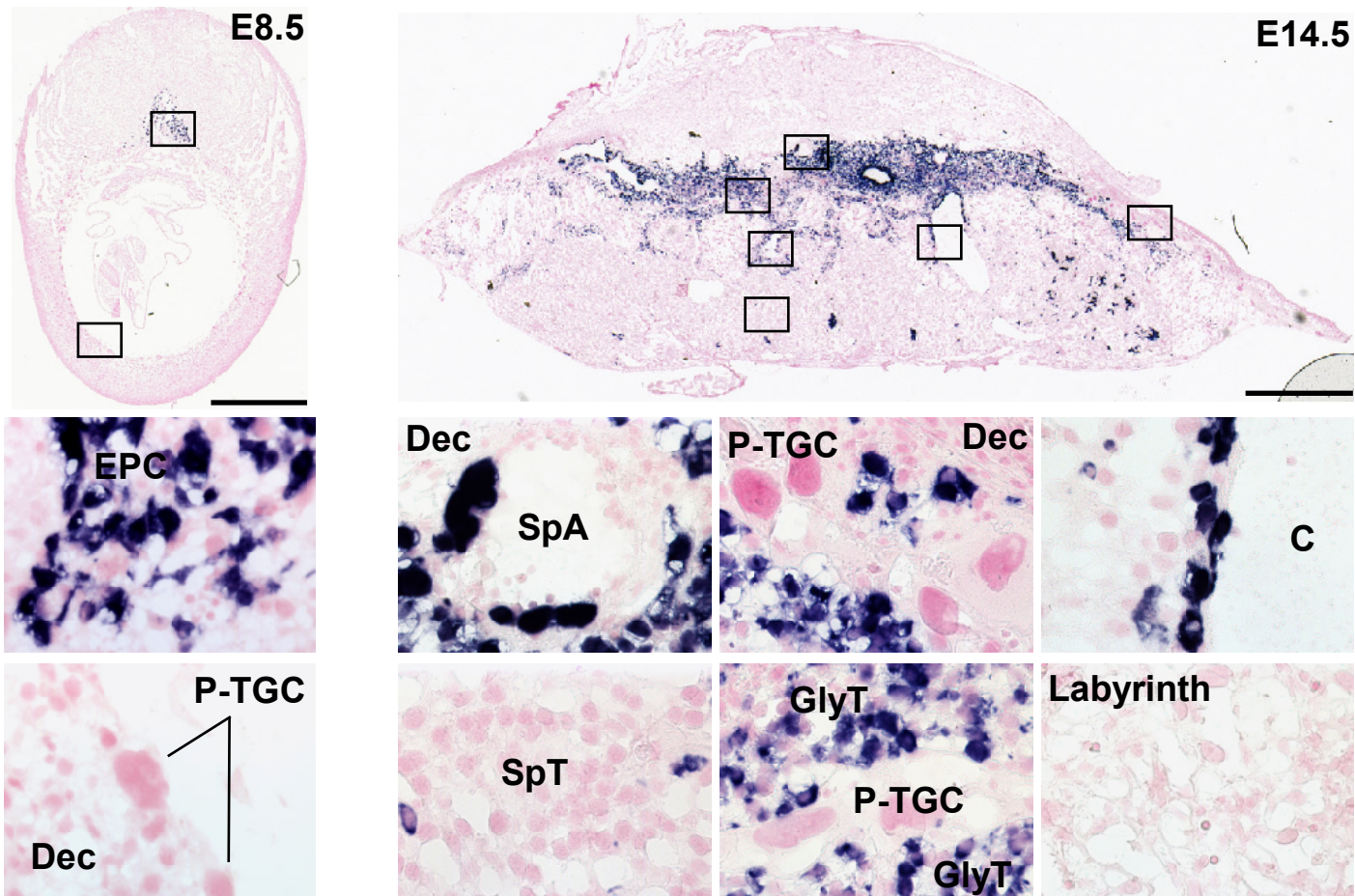

B

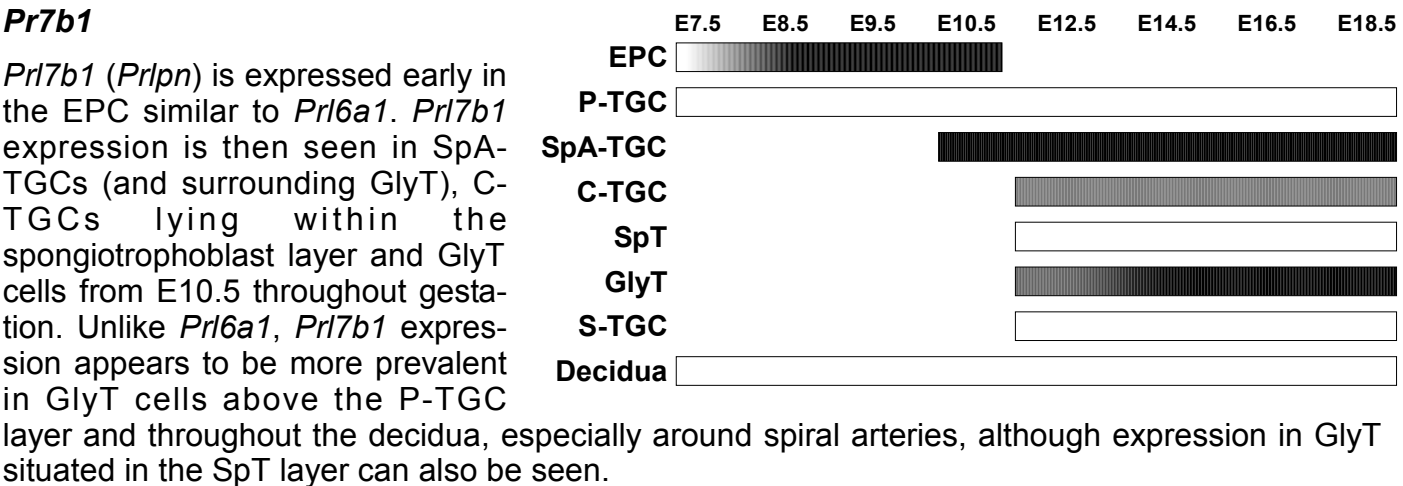

Previous publications showing mouse *Prl7b1* expression: (Wiemers et al., 2003).

Supplement: Additional file 13 — A – In situ hybridizations of early (E8.5) and mid to late gestation (E12.5, E14.5, or E18.5) placenta for each member of the PRL/PL family. Higher magnifications emphasize particular trophoblast subtypes including parietal TGCs, spiral artery TGCs, canal TGCs, sinusoidal TGCs, spongiotrophoblast, glycogen trophoblast cells, and decidua. B – Temporal gene expression data (based in situ hybridization signals) for individual placental cell types. Shades of grey depict an estimation of the percentage of each cell type that expresses the gene. White – 0%, Light grey ~25%, Medium Grey ~50%, Dark grey ~75%, Black > 75%. Summary of in situ hybridization data for Prl7b1. [file 1471-2164-9-352-S13.pdf]
