# Supplementary material for: Spatial and temporal expression of the 23 murine Prolactin/Placental Lactogen-related genes is not associated with their position in the locus
Source: BMC Genomics. 2008 Jul 28;9:352. doi: 10.1186/1471-2164-9-352 (PMC2527339; doi:10.1186/1471-2164-9-352)
Supplement: Additional file 4 — A – In situ hybridizations of early (E8.5) and mid to late gestation (E12.5, E14.5, or E18.5) placenta for each member of the PRL/PL family. Higher magnifications emphasize particular trophoblast subtypes including parietal TGCs, spiral artery TGCs, canal TGCs, sinusoidal TGCs, spongiotrophoblast, glycogen trophoblast cells, and decidua. B – Temporal gene expression data (based in situ hybridization signals) for individual placental cell types. Shades of grey depict an estimation of the percentage of each cell type that expresses the gene. White – 0%, Light grey ~25%, Medium Grey ~50%, Dark grey ~75%, Black > 75%. Summary of in situ hybridization data for Prl3b1. [file 1471-2164-9-352-S4.pdf]

Gene: *Prl3b1* (*Csh2/Pl2*)

A

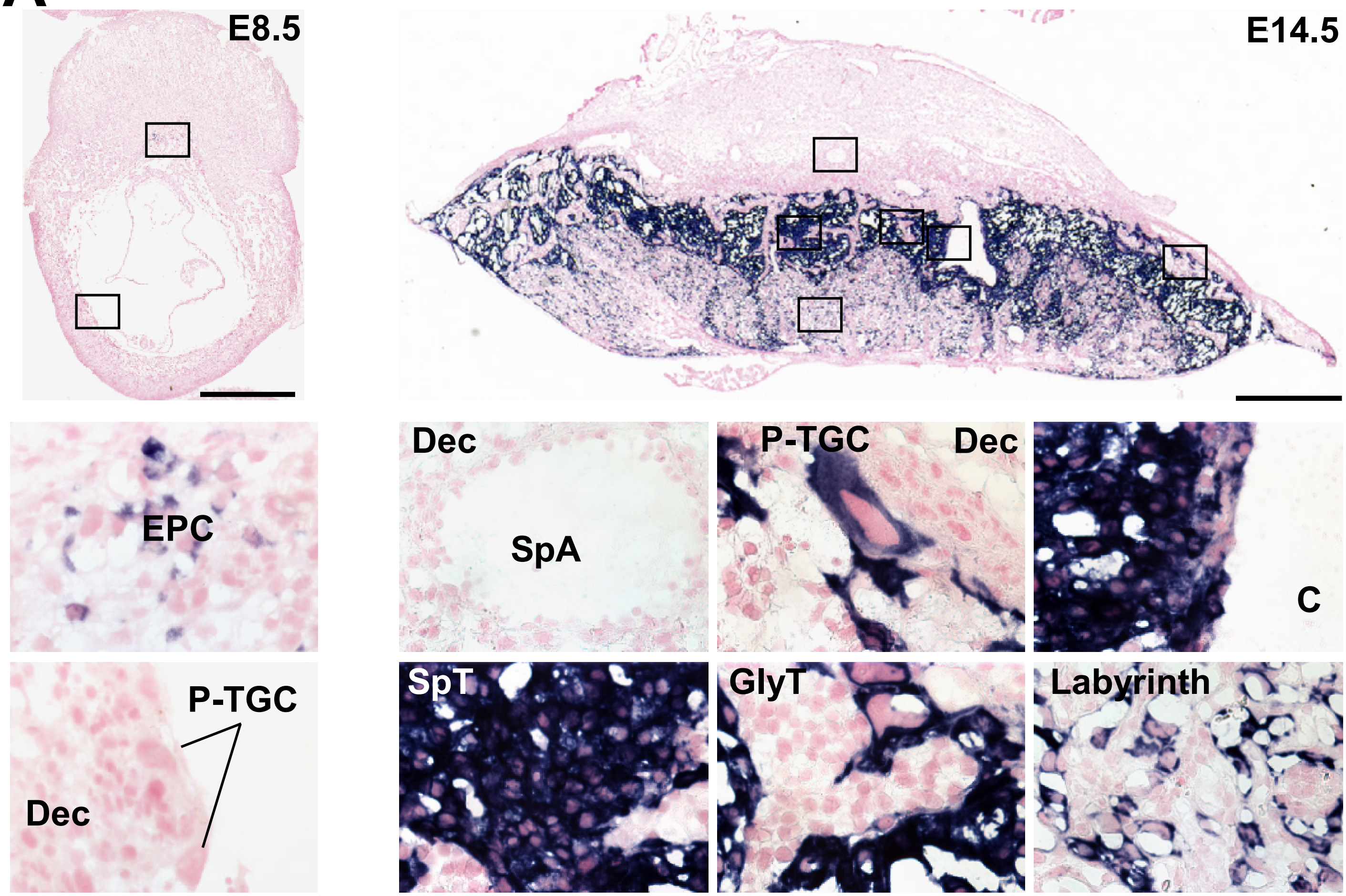

B

Prl3b1

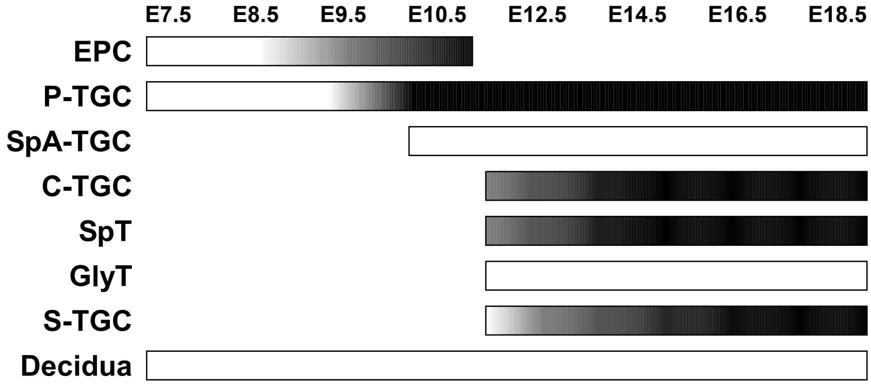

Placental lactogen 2 (*Prl3b1*) is expressed by P-TGCs after *Prl3d* expression begins to decline around E9.5 (Carney et al., 1993), and has traditionally been used as a marker of TGC formation along with *Prl3d* (*Pl1*) and *Prl2c* (*Plf*). However, *Prl3b1* is also expressed in spongiotrophoblast (SpT) beginning around E9.5-10.5. In addition, some expression of *Prl3b1* can be detected in the ectoplacental cone (EPC) as early as E8.5. While *Prl3b1* expression cannot be detected in TGCs which invade the spiral arteries (SpA-TGCs), expression can be seen in TGCs lining the maternal canals (C-TGCs). *Prl3b1* is one of the few prolactin family members which is expressed in TGCs of the labyrinth layer; that is the TGCs lining the maternal sinusoids, previously referred to as mononuclear trophoblast but now called sinusoidal trophoblast giant cells (S-TGCs).
