# Supplementary material for: Spatial and temporal expression of the 23 murine Prolactin/Placental Lactogen-related genes is not associated with their position in the locus
Source: BMC Genomics. 2008 Jul 28;9:352. doi: 10.1186/1471-2164-9-352 (PMC2527339; doi:10.1186/1471-2164-9-352)
Supplement: Additional file 6 — A – In situ hybridizations of early (E8.5) and mid to late gestation (E12.5, E14.5, or E18.5) placenta for each member of the PRL/PL family. Higher magnifications emphasize particular trophoblast subtypes including parietal TGCs, spiral artery TGCs, canal TGCs, sinusoidal TGCs, spongiotrophoblast, glycogen trophoblast cells, and decidua. B – Temporal gene expression data (based in situ hybridization signals) for individual placental cell types. Shades of grey depict an estimation of the percentage of each cell type that expresses the gene. White – 0%, Light grey ~25%, Medium Grey ~50%, Dark grey ~75%, Black > 75%. Summary of in situ hybridization data for Prl6a1. [file 1471-2164-9-352-S6.pdf]

Gene: *Prl6a1* (*Prlpb*)

A

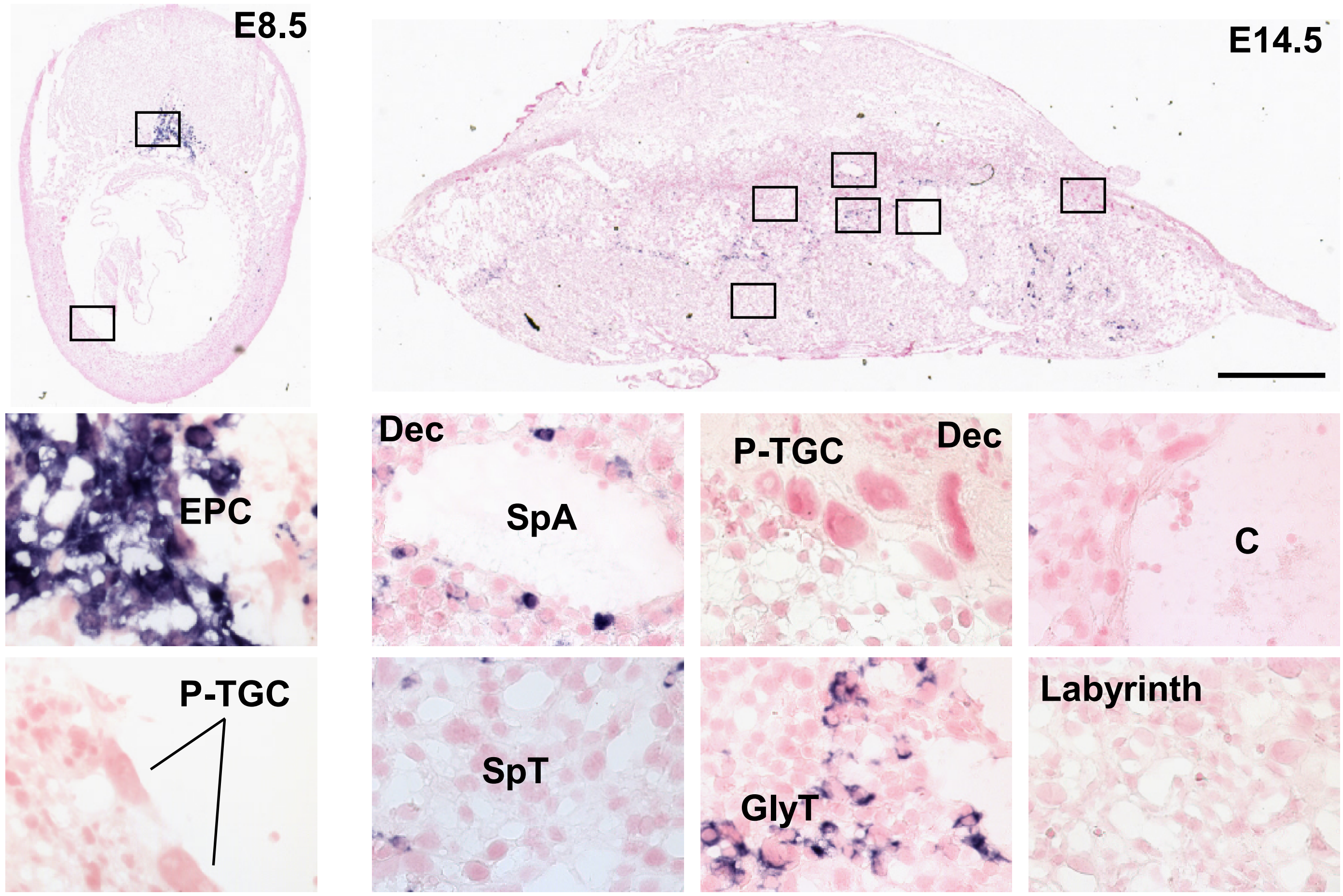

B

Prl6a1

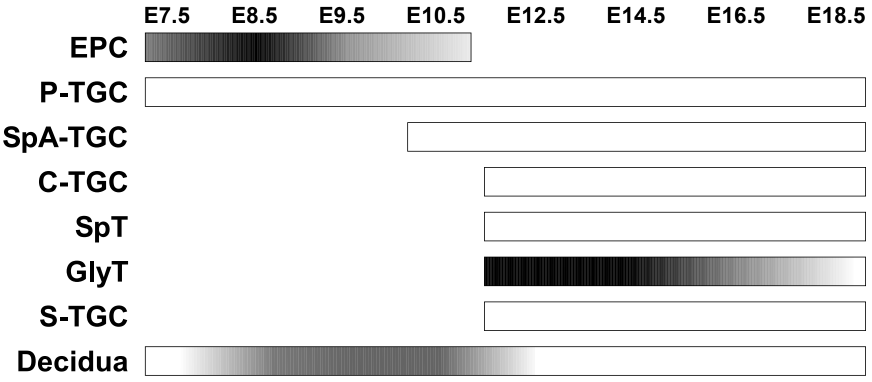

Prl6a1 is expressed early in the EPC as well as the antimesometrial decidua. Mesometrial decidual expression has not been observed. Later in gestation, with the formation of the spongiotrophoblast/glycogen trophpblast layer around E10.5, it is clear that *Prl6a1* expression is restricted to GlyT cells. By E10.5 *Prl6a1* expression is not uniform throughout the GlyT population, while some *Prl6a1* (+) GlyT are seen above the TGC layer in the decidua and in proximity to spiral arteries, most *Prl6a1* (+) GlyT appear to be confined within the spongiotrophoblast layer itself. This is complementary to *Prl7b1* expression for example, which appears to be predominantly expressed in GlyT cells located above the TGC layer and within the maternal decidua, although there is clearly overlap of *Prl6a1* and *Prl7b1* expression in many cells. By E16.5, very few cells still express *Prl6a1*. Previous publications showing mouse *Prl6a1* expression: (Lin et al., 1997a; Muller et al., 1998).
