# Supplementary material for: Spatial and temporal expression of the 23 murine Prolactin/Placental Lactogen-related genes is not associated with their position in the locus
Source: BMC Genomics. 2008 Jul 28;9:352. doi: 10.1186/1471-2164-9-352 (PMC2527339; doi:10.1186/1471-2164-9-352)
Supplement: Additional file 7 — A – In situ hybridizations of early (E8.5) and mid to late gestation (E12.5, E14.5, or E18.5) placenta for each member of the PRL/PL family. Higher magnifications emphasize particular trophoblast subtypes including parietal TGCs, spiral artery TGCs, canal TGCs, sinusoidal TGCs, spongiotrophoblast, glycogen trophoblast cells, and decidua. B – Temporal gene expression data (based in situ hybridization signals) for individual placental cell types. Shades of grey depict an estimation of the percentage of each cell type that expresses the gene. White – 0%, Light grey ~25%, Medium Grey ~50%, Dark grey ~75%, Black > 75%. Summary of in situ hybridization data for Prl8a2. [file 1471-2164-9-352-S7.pdf]

Gene: *Prl8a2* (*Dtprp*)

A

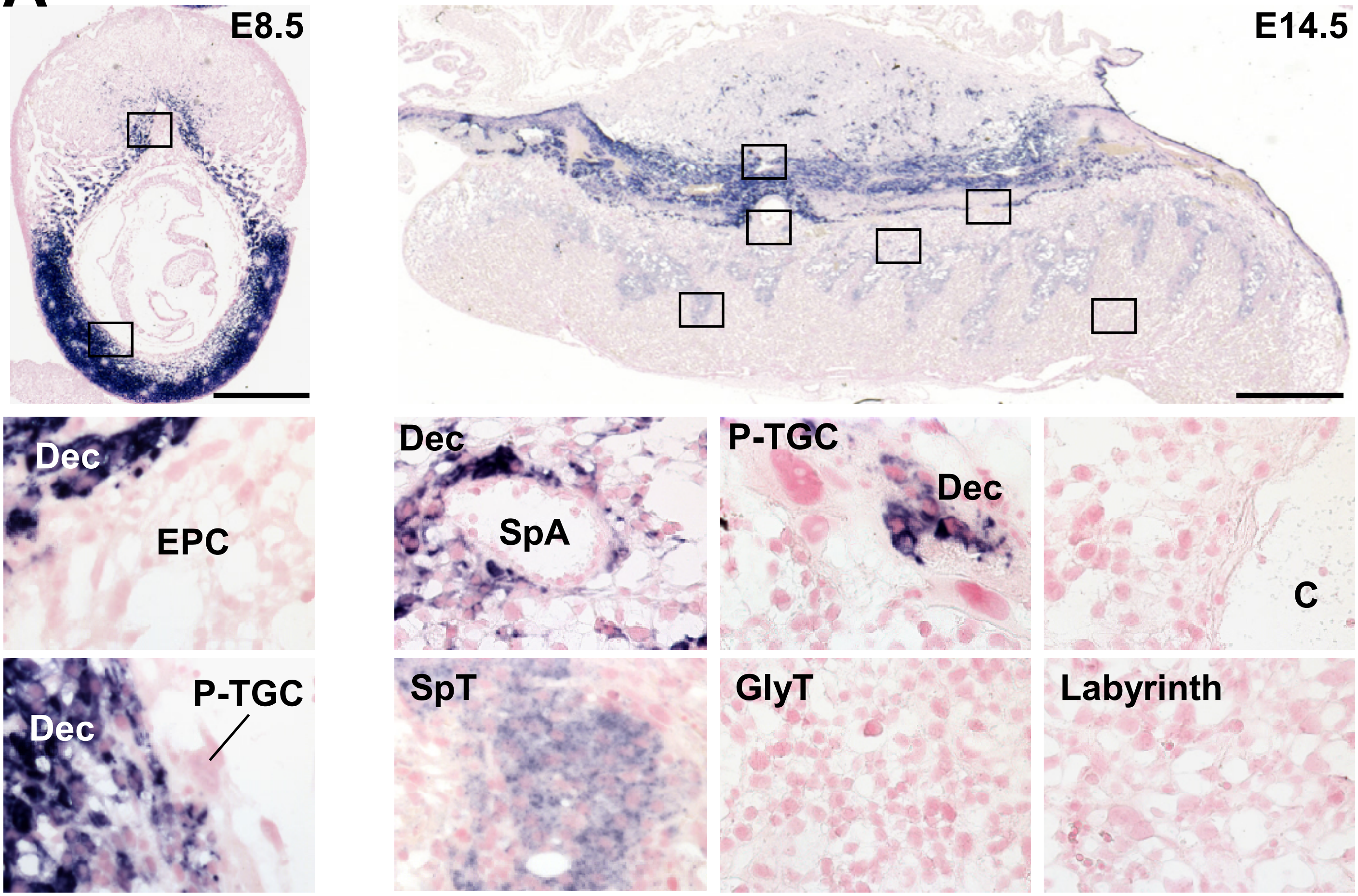

B

Prl8a2

Prl8a2 (also called *D/tPrp*, *Dprp* or *Mdprp*) is expressed exclusively in decidual cells surrounding the implanting conceptus. Expression can be detected early in the antimesometrial decidua and later, to a lesser extent, the mesometrial decidua. Northern blot data in the current study is somewhat misleading for the expression patterns for *Prl8a2* as the earliest sample, E8.5, does not contain any decidua and samples taken after E10.5 contain very little decidua due to the thinning of the layer and the dissection technique. Therefore expression appears to be strongest at E9.5 and E10.5. In fact, expression in the antimesometrial compartment is very strong at E7.5 and E8.5 as well. Also, mesometrial expression of *Prl8a2* later in gestation is not uniform, with strongest expression immediately in contact with trophoblast tissue and around spiral arteries. In mid-gestation *Prl8a2* expression can also be seen in SpT.

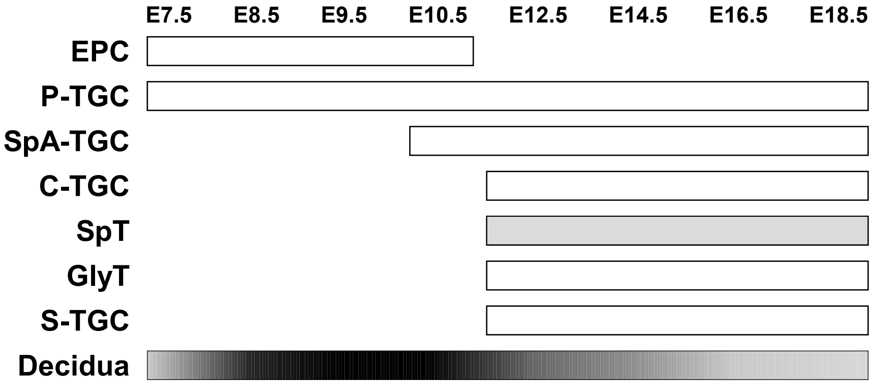

Previous publications showing mouse *Prl8a2* expression: (Lin et al., 1997a; Alam et al., 2007).
