# Supplementary material for: Spatial and temporal expression of the 23 murine Prolactin/Placental Lactogen-related genes is not associated with their position in the locus
Source: BMC Genomics. 2008 Jul 28;9:352. doi: 10.1186/1471-2164-9-352 (PMC2527339; doi:10.1186/1471-2164-9-352)
Supplement: Additional file 8 — A – In situ hybridizations of early (E8.5) and mid to late gestation (E12.5, E14.5, or E18.5) placenta for each member of the PRL/PL family. Higher magnifications emphasize particular trophoblast subtypes including parietal TGCs, spiral artery TGCs, canal TGCs, sinusoidal TGCs, spongiotrophoblast, glycogen trophoblast cells, and decidua. B – Temporal gene expression data (based in situ hybridization signals) for individual placental cell types. Shades of grey depict an estimation of the percentage of each cell type that expresses the gene. White – 0%, Light grey ~25%, Medium Grey ~50%, Dark grey ~75%, Black > 75%. Summary of in situ hybridization data for Prl2b1. [file 1471-2164-9-352-S8.pdf]

Gene: *Prl2b1* (*Prlpk*)

A

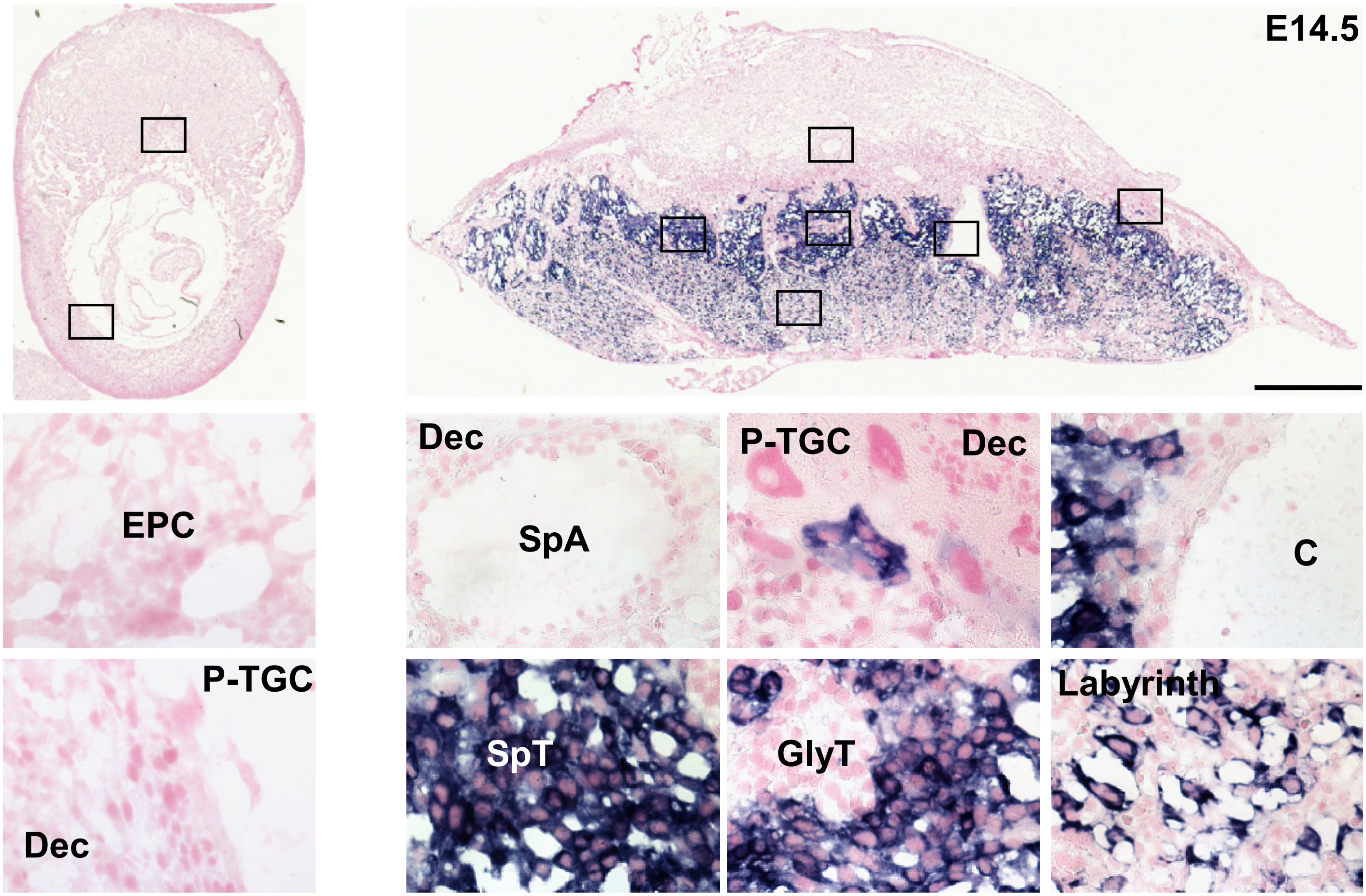

B

Prl2b1

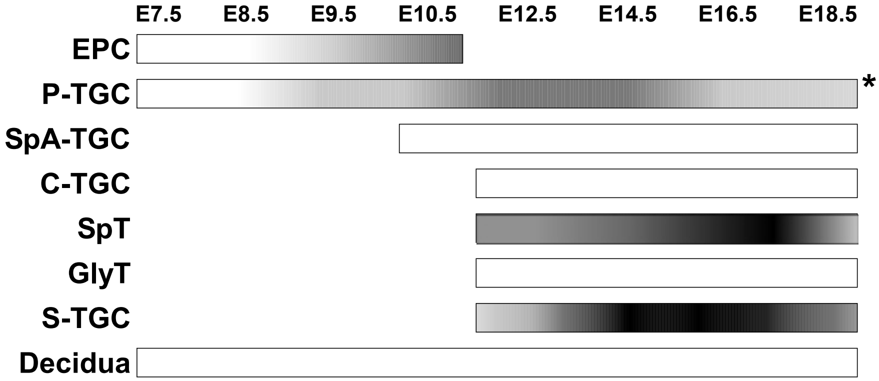

Prl2b1 expression is very similar to *Prl3b1*(*Csh2*/*Pl2*), with some notable exceptions. *Prl2b1* expression is not expressed in the EPC early in gestation. *Expression in some P-TGCs, although only in a small subset and only in secondary P-TGCs, is evident after E9.5. *Prl2b1* is expressed mainly in SpT and S-TGCs beginning after E10.5 and continuing throughout gestation. GlyT cells are negative for *Prl2b1* expression. In contrast to *Prl3b1*, C-TGCs clearly do NOT express *Prl2b1*.

Previous publications showing mouse *Prl2b1* expression: (Dai et al., 2000; Wiemers et al., 2003).
