# Supplementary material for: Spatial and temporal expression of the 23 murine Prolactin/Placental Lactogen-related genes is not associated with their position in the locus
Source: BMC Genomics. 2008 Jul 28;9:352. doi: 10.1186/1471-2164-9-352 (PMC2527339; doi:10.1186/1471-2164-9-352)

# Gene: *Prl8a8* (*Prlpc3*)

**A**

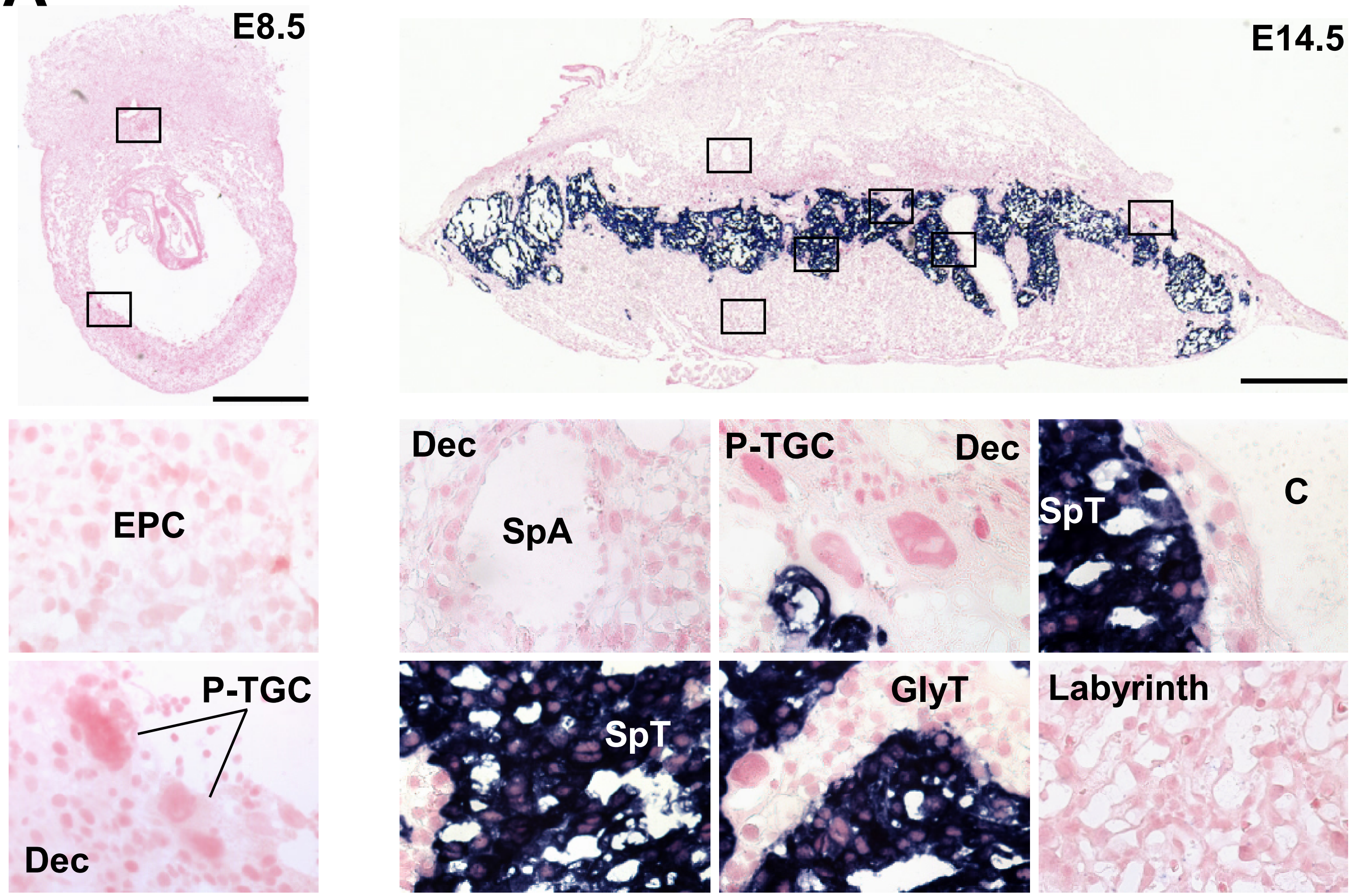


**B**

### *Prl8a8*

*Prl8a8* (*Prlpc3*), also known as *Prlpcγ*, is expressed exclusively in SpT cells beginning around E11.5 and continuing throughout gestation.

Previous publications showing mouse *Prl8a8* expression: (Wiemers et al., 2003).

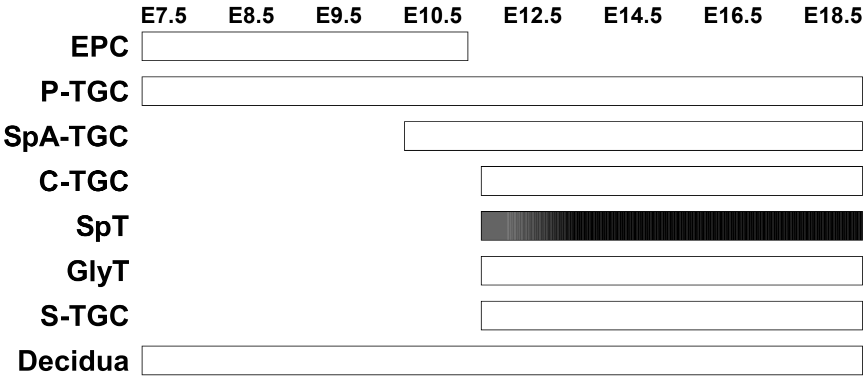

Supplement: Additional file 10 — A – In situ hybridizations of early (E8.5) and mid to late gestation (E12.5, E14.5, or E18.5) placenta for each member of the PRL/PL family. Higher magnifications emphasize particular trophoblast subtypes including parietal TGCs, spiral artery TGCs, canal TGCs, sinusoidal TGCs, spongiotrophoblast, glycogen trophoblast cells, and decidua. B – Temporal gene expression data (based in situ hybridization signals) for individual placental cell types. Shades of grey depict an estimation of the percentage of each cell type that expresses the gene. White – 0%, Light grey ~25%, Medium Grey ~50%, Dark grey ~75%, Black > 75%. Summary of in situ hybridization data for Prl8a8. [file 1471-2164-9-352-S10.pdf]
