# Supplementary material for: Spatial and temporal expression of the 23 murine Prolactin/Placental Lactogen-related genes is not associated with their position in the locus
Source: BMC Genomics. 2008 Jul 28;9:352. doi: 10.1186/1471-2164-9-352 (PMC2527339; doi:10.1186/1471-2164-9-352)
Supplement: Additional file 11 — A – In situ hybridizations of early (E8.5) and mid to late gestation (E12.5, E14.5, or E18.5) placenta for each member of the PRL/PL family. Higher magnifications emphasize particular trophoblast subtypes including parietal TGCs, spiral artery TGCs, canal TGCs, sinusoidal TGCs, spongiotrophoblast, glycogen trophoblast cells, and decidua. B – Temporal gene expression data (based in situ hybridization signals) for individual placental cell types. Shades of grey depict an estimation of the percentage of each cell type that expresses the gene. White – 0%, Light grey ~25%, Medium Grey ~50%, Dark grey ~75%, Black > 75%. Summary of in situ hybridization data for Prl8a9. [file 1471-2164-9-352-S11.pdf]

Gene: *Prl8a9* (*Prlpc2*)

A

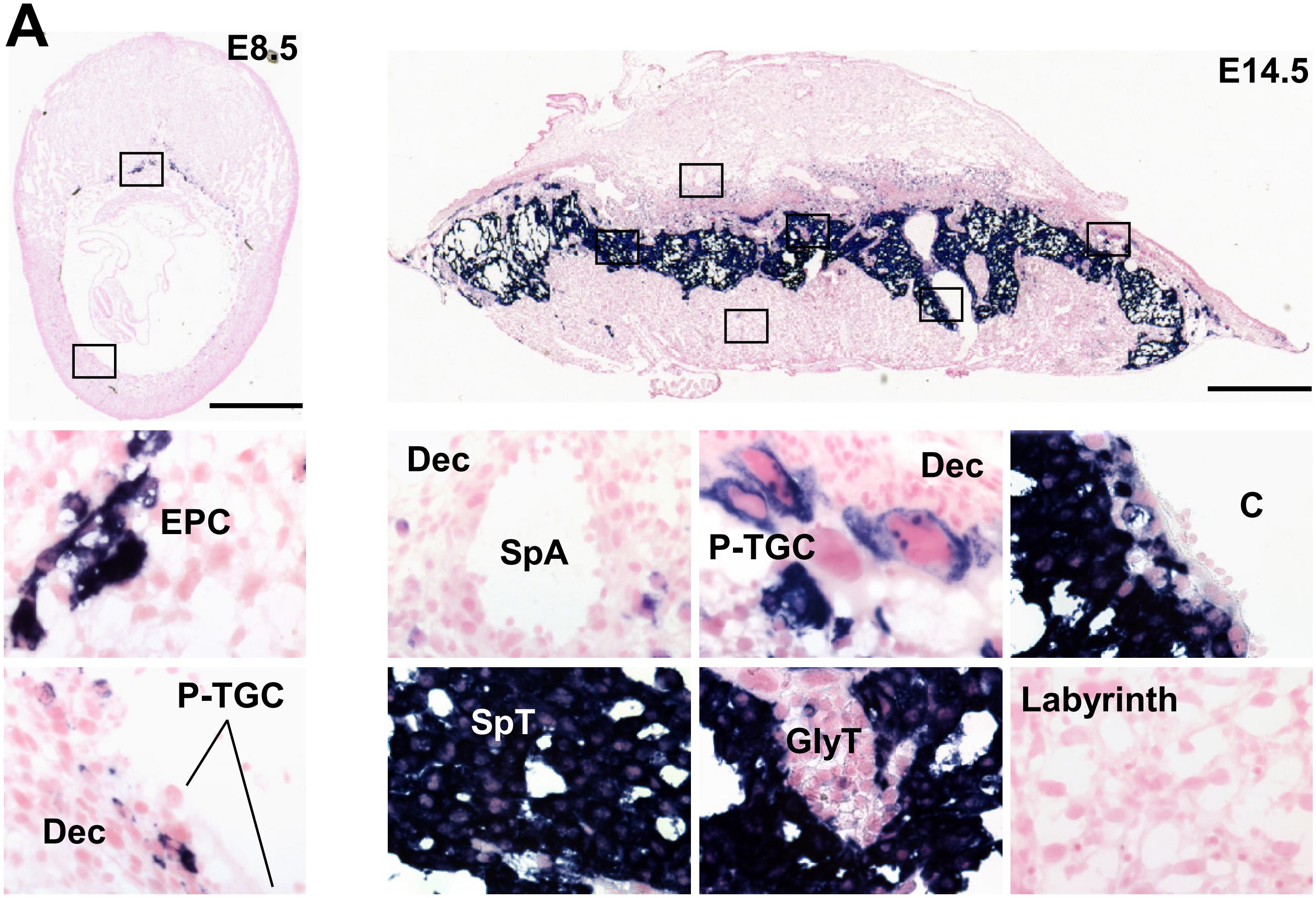

B

Prl8a9

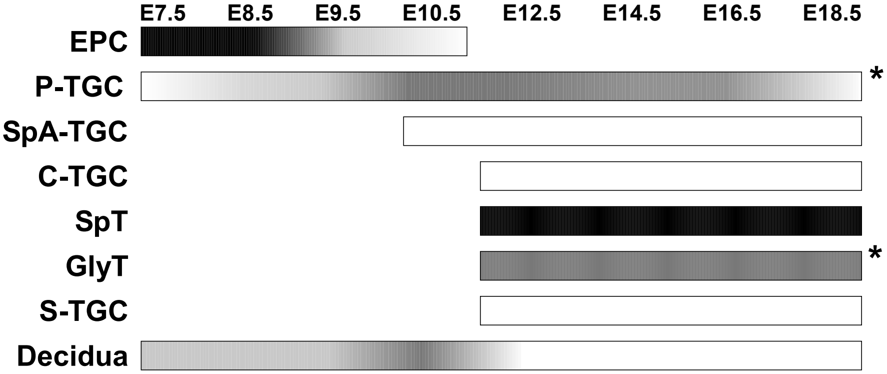

Prl8a9 (*Prlpc2*), also known as *Prlpc*β, is expressed initially in the decidua surrounding the implantation site at E7.5. Decidual expression persists, at least antimesometrially, until approximately E10.5. Strong expression in the EPC is also evident early in gestation as well as in a subset of P-TGCs. *Interestingly, only very few P-TGCs throughout gestation were *Prl8a9* (+) in the CD1 background, while considerably more *Prl8a9* (+) P-TGCs were seen in the C57/B6 and 129svj backgrounds. After E10.5 the most significant levels of *Prl8a9* expression were seen in SpT where expression continues at very high levels until term. Longer in situ hybridization exposure times also revealed GlyT expression, albeit at much lower levels than seen in SpT.

Previous publications showing mouse *Prl8a9* expression: (Hwang et al., 2000).
