# Supplementary material for: Spatial and temporal expression of the 23 murine Prolactin/Placental Lactogen-related genes is not associated with their position in the locus
Source: BMC Genomics. 2008 Jul 28;9:352. doi: 10.1186/1471-2164-9-352 (PMC2527339; doi:10.1186/1471-2164-9-352)
Supplement: Additional file 12 — A – In situ hybridizations of early (E8.5) and mid to late gestation (E12.5, E14.5, or E18.5) placenta for each member of the PRL/PL family. Higher magnifications emphasize particular trophoblast subtypes including parietal TGCs, spiral artery TGCs, canal TGCs, sinusoidal TGCs, spongiotrophoblast, glycogen trophoblast cells, and decidua. B – Temporal gene expression data (based in situ hybridization signals) for individual placental cell types. Shades of grey depict an estimation of the percentage of each cell type that expresses the gene. White – 0%, Light grey ~25%, Medium Grey ~50%, Dark grey ~75%, Black > 75%. Summary of in situ hybridization data for Prl8a1. [file 1471-2164-9-352-S12.pdf]

Gene: *Prl8a1* (*Prlpc4*)

A

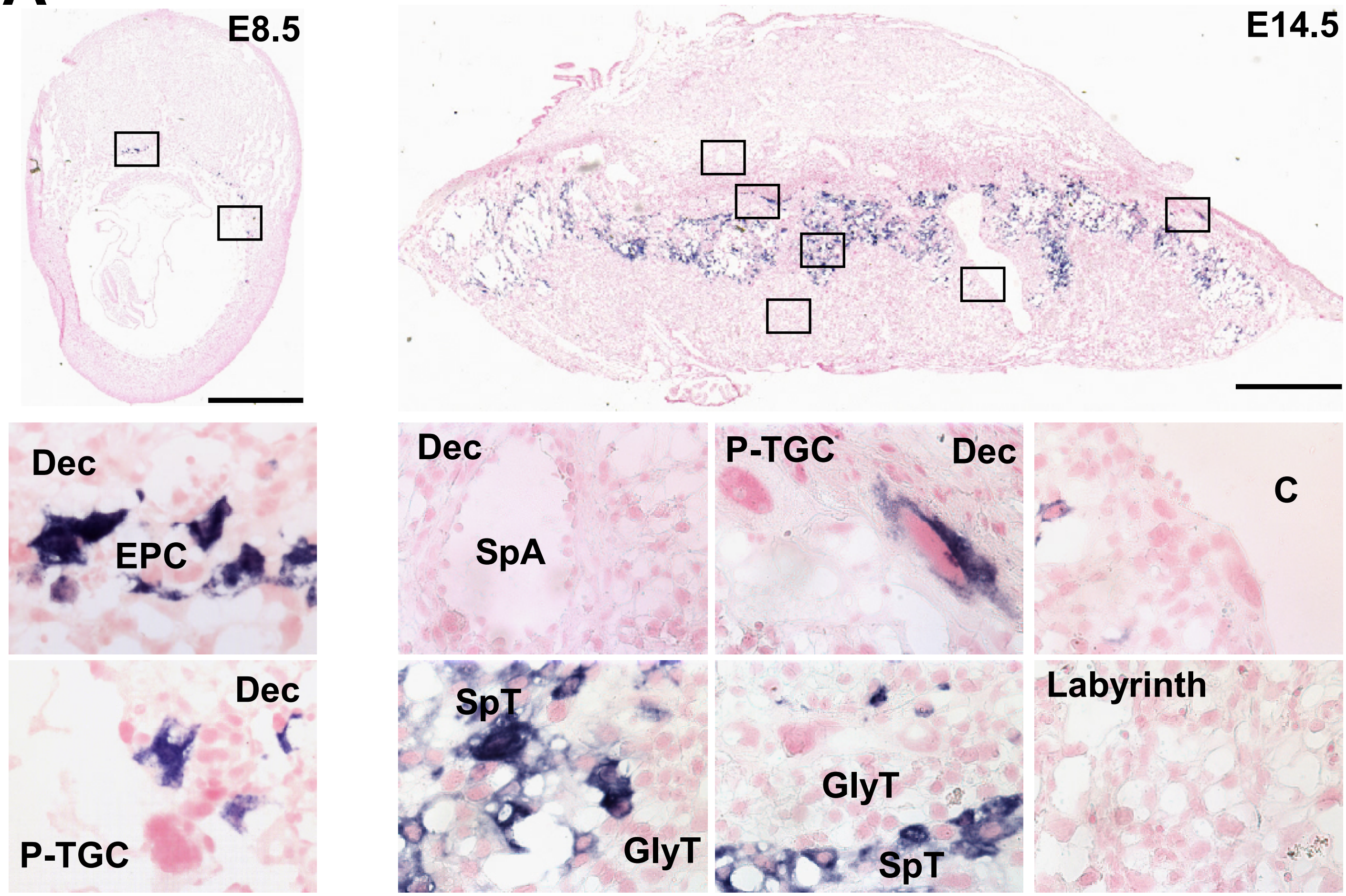

B

Prl8a1

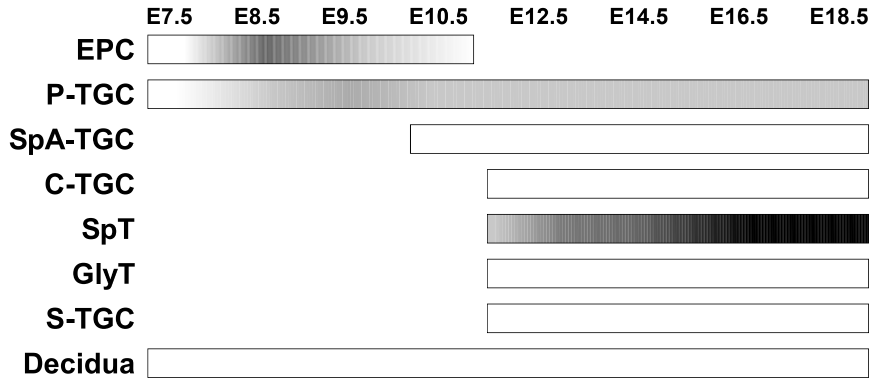

Prl8a1 (*Prlpc4*), also known as *Prlpcδ*, is expressed early in the EPC region in P-TGCs on the edge of the cone. Also, a subset of primary P-TGCs express *Prl8a1*. Expression of *Prl8a1* in some P-TGCs persists throughout gestation. *Prl8a1* expression begins within SpT cells as early as E10.5 and is maintained throughout pregnancy. It is important to note that *Prl8a1* expression within the SpT cells is not uniform as for *Prl8a9* or *Prl8a8*, but appears in a subset of SpT cells.

Previous publications showing mouse *Prl8a1* expression: (Wiemers et al., 2003).

Note: we did not see expression of *Prl8a1* in GlyT (or migratory trophoblast) or as robust expression within SpT as shown in this paper. Discrepancies could be the result of differences in in situ hybridization technique/sensitivity or gestational age used (E19.5 in Wiemers paper).
