# Supplementary material for: Spatial and temporal expression of the 23 murine Prolactin/Placental Lactogen-related genes is not associated with their position in the locus
Source: BMC Genomics. 2008 Jul 28;9:352. doi: 10.1186/1471-2164-9-352 (PMC2527339; doi:10.1186/1471-2164-9-352)

Gene: *Prl7a1* (*Prlpe*)

A

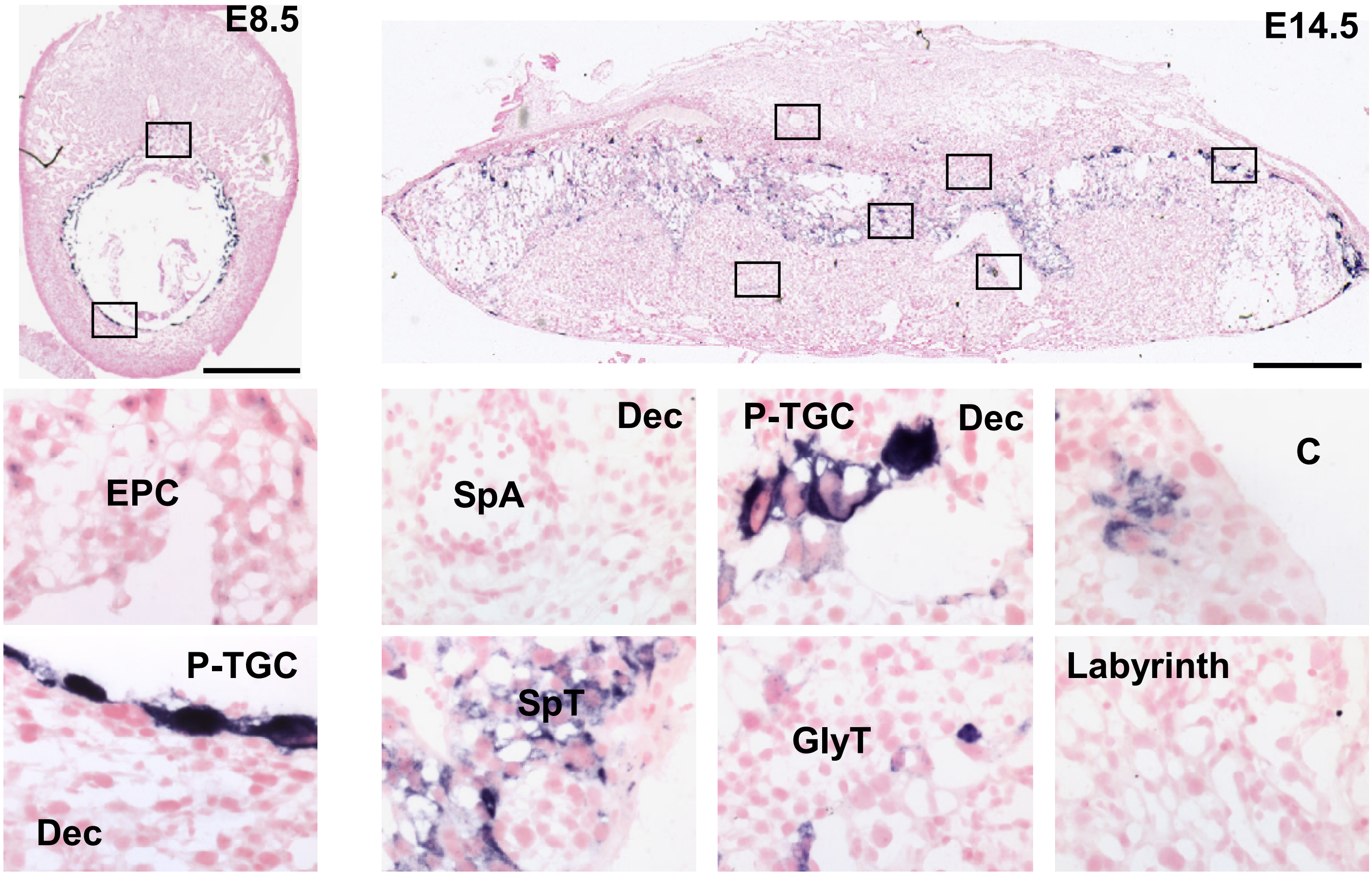

B

Prl7a1

Prl7a1 is expressed in most, but not all, P-TGCs early in gestation, including primary P-TGCs. While *Prlpe* expression can be seen in P-TGCs throughout gestation, expression is significantly reduced by E14.5. In addition, SpT expression of *Prlpe* can be seen by E12.5, although only a subset of SpT.

Previous publications showing mouse *Prl7a1* expression: (Lin et al., 1997b).

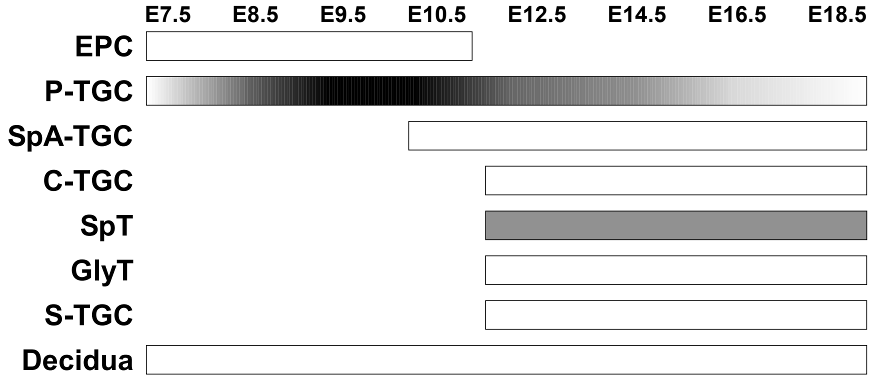

Supplement: Additional file 14 — A – In situ hybridizations of early (E8.5) and mid to late gestation (E12.5, E14.5, or E18.5) placenta for each member of the PRL/PL family. Higher magnifications emphasize particular trophoblast subtypes including parietal TGCs, spiral artery TGCs, canal TGCs, sinusoidal TGCs, spongiotrophoblast, glycogen trophoblast cells, and decidua. B – Temporal gene expression data (based in situ hybridization signals) for individual placental cell types. Shades of grey depict an estimation of the percentage of each cell type that expresses the gene. White – 0%, Light grey ~25%, Medium Grey ~50%, Dark grey ~75%, Black > 75%. Summary of in situ hybridization data for Prl7a1. [file 1471-2164-9-352-S14.pdf]
