# Supplementary material for: Spatial and temporal expression of the 23 murine Prolactin/Placental Lactogen-related genes is not associated with their position in the locus
Source: BMC Genomics. 2008 Jul 28;9:352. doi: 10.1186/1471-2164-9-352 (PMC2527339; doi:10.1186/1471-2164-9-352)

Gene: *Prl7a2* (*Prlpf*)

A

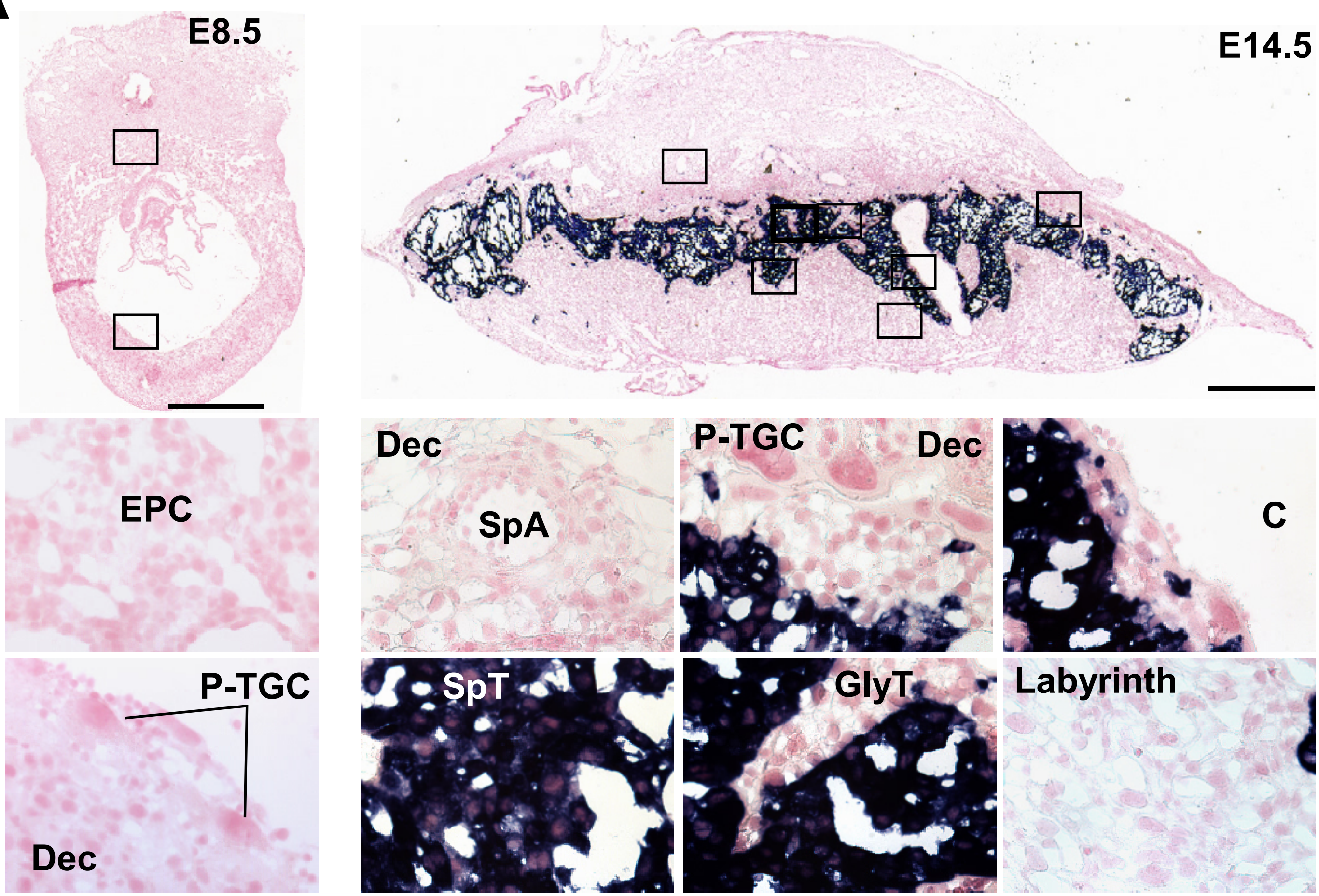

B

Prl7a2

Prl7a2 (*Prlpf*) is highly expressed in SpT starting after E10.5 and continuing throughout gestation. In addition, the occasional *Prl7a2*⁺ P-TGC can be detected after E9.5.

Previous publications showing mouse *Prl7a2* expression: (Lin et al., 1997b).

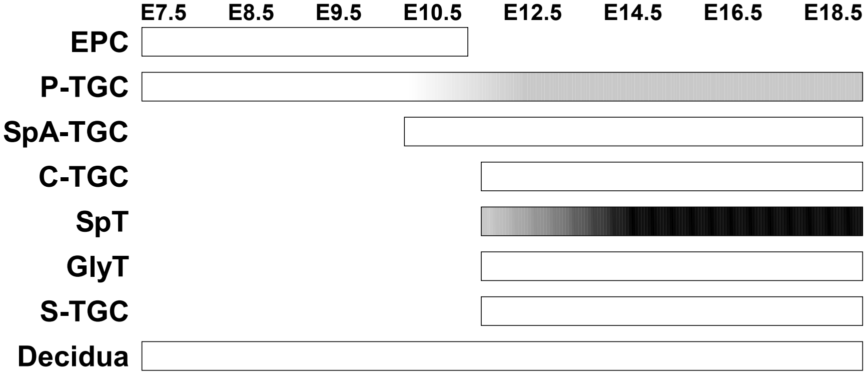

Supplement: Additional file 15 — A – In situ hybridizations of early (E8.5) and mid to late gestation (E12.5, E14.5, or E18.5) placenta for each member of the PRL/PL family. Higher magnifications emphasize particular trophoblast subtypes including parietal TGCs, spiral artery TGCs, canal TGCs, sinusoidal TGCs, spongiotrophoblast, glycogen trophoblast cells, and decidua. B – Temporal gene expression data (based in situ hybridization signals) for individual placental cell types. Shades of grey depict an estimation of the percentage of each cell type that expresses the gene. White – 0%, Light grey ~25%, Medium Grey ~50%, Dark grey ~75%, Black > 75%. Summary of in situ hybridization data for Prl7a2. [file 1471-2164-9-352-S15.pdf]
