# Supplementary material for: Spatial and temporal expression of the 23 murine Prolactin/Placental Lactogen-related genes is not associated with their position in the locus
Source: BMC Genomics. 2008 Jul 28;9:352. doi: 10.1186/1471-2164-9-352 (PMC2527339; doi:10.1186/1471-2164-9-352)
Supplement: Additional file 16 — A – In situ hybridizations of early (E8.5) and mid to late gestation (E12.5, E14.5, or E18.5) placenta for each member of the PRL/PL family. Higher magnifications emphasize particular trophoblast subtypes including parietal TGCs, spiral artery TGCs, canal TGCs, sinusoidal TGCs, spongiotrophoblast, glycogen trophoblast cells, and decidua. B – Temporal gene expression data (based in situ hybridization signals) for individual placental cell types. Shades of grey depict an estimation of the percentage of each cell type that expresses the gene. White – 0%, Light grey ~25%, Medium Grey ~50%, Dark grey ~75%, Black > 75%. Summary of in situ hybridization data for Prl7d1. [file 1471-2164-9-352-S16.pdf]

Gene: *Prl7d1* (*Plfr/Prp*)

A

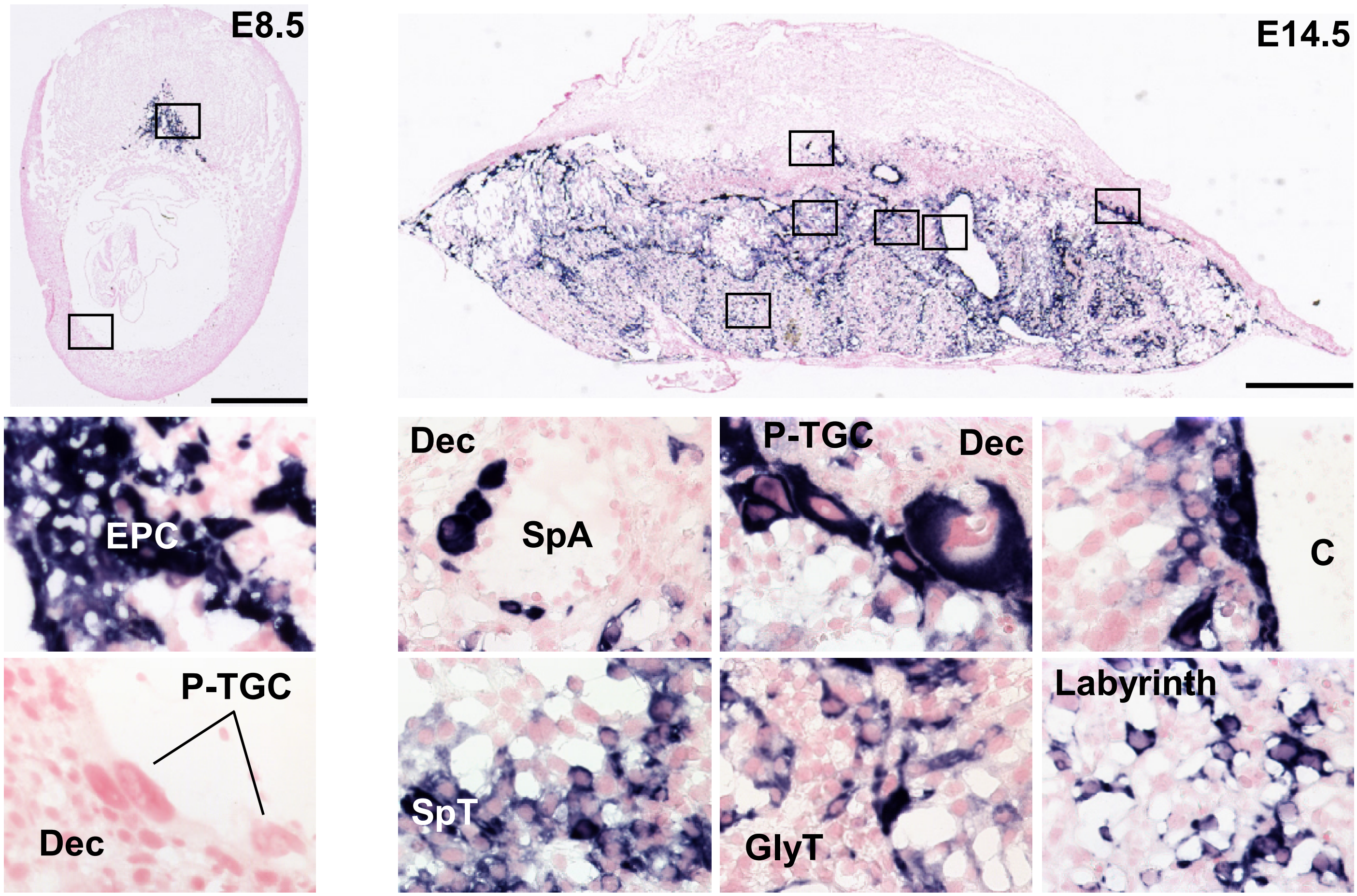

B

Prl7d1

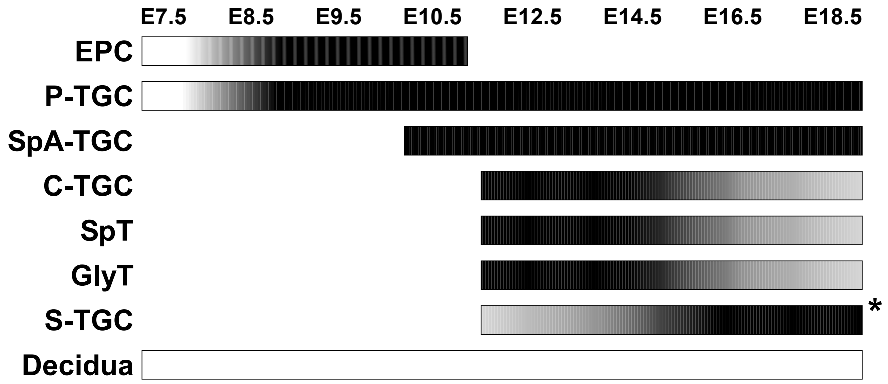

Prl7d1 (*Plfr*) is perhaps the most widely expressed prolactin family member. Expression of *Prl7d1* can be detected early in the EPC and later in many, but not all, P-TGCs. *Prl7d1* is expressed by SpA-TGCs and the glycogen trophoblast cells (GlyT) which accumulate around the arteries throughout gestation. Continuous with the spiral arteries, the maternal blood canals, lined by C-TGCs, and maternal sinusoids, lined by S-TGCs, all express *Prl7d1*. In addition, both SpT and GlyT express *Prl7d1*, although not all of each subtype. * Interestingly, in both CD1 and C57/B6 mice *Plfr* expression is dramatically reduced by E16.5 in SpT and GlyT and is almost entirely absent from S-TGC at this stage, but in 129svj mice, *Plfr* expression persists at these later stages, particularly in S-TGCs.
