# Supplementary material for: Spatial and temporal expression of the 23 murine Prolactin/Placental Lactogen-related genes is not associated with their position in the locus
Source: BMC Genomics. 2008 Jul 28;9:352. doi: 10.1186/1471-2164-9-352 (PMC2527339; doi:10.1186/1471-2164-9-352)
Supplement: Additional file 17 — A – In situ hybridizations of early (E8.5) and mid to late gestation (E12.5, E14.5, or E18.5) placenta for each member of the PRL/PL family. Higher magnifications emphasize particular trophoblast subtypes including parietal TGCs, spiral artery TGCs, canal TGCs, sinusoidal TGCs, spongiotrophoblast, glycogen trophoblast cells, and decidua. B – Temporal gene expression data (based in situ hybridization signals) for individual placental cell types. Shades of grey depict an estimation of the percentage of each cell type that expresses the gene. White – 0%, Light grey ~25%, Medium Grey ~50%, Dark grey ~75%, Black > 75%. Summary of in situ hybridization data for Prl7c1. [file 1471-2164-9-352-S17.pdf]

Gene: *Prl7c1* (*Prlpo*)

A

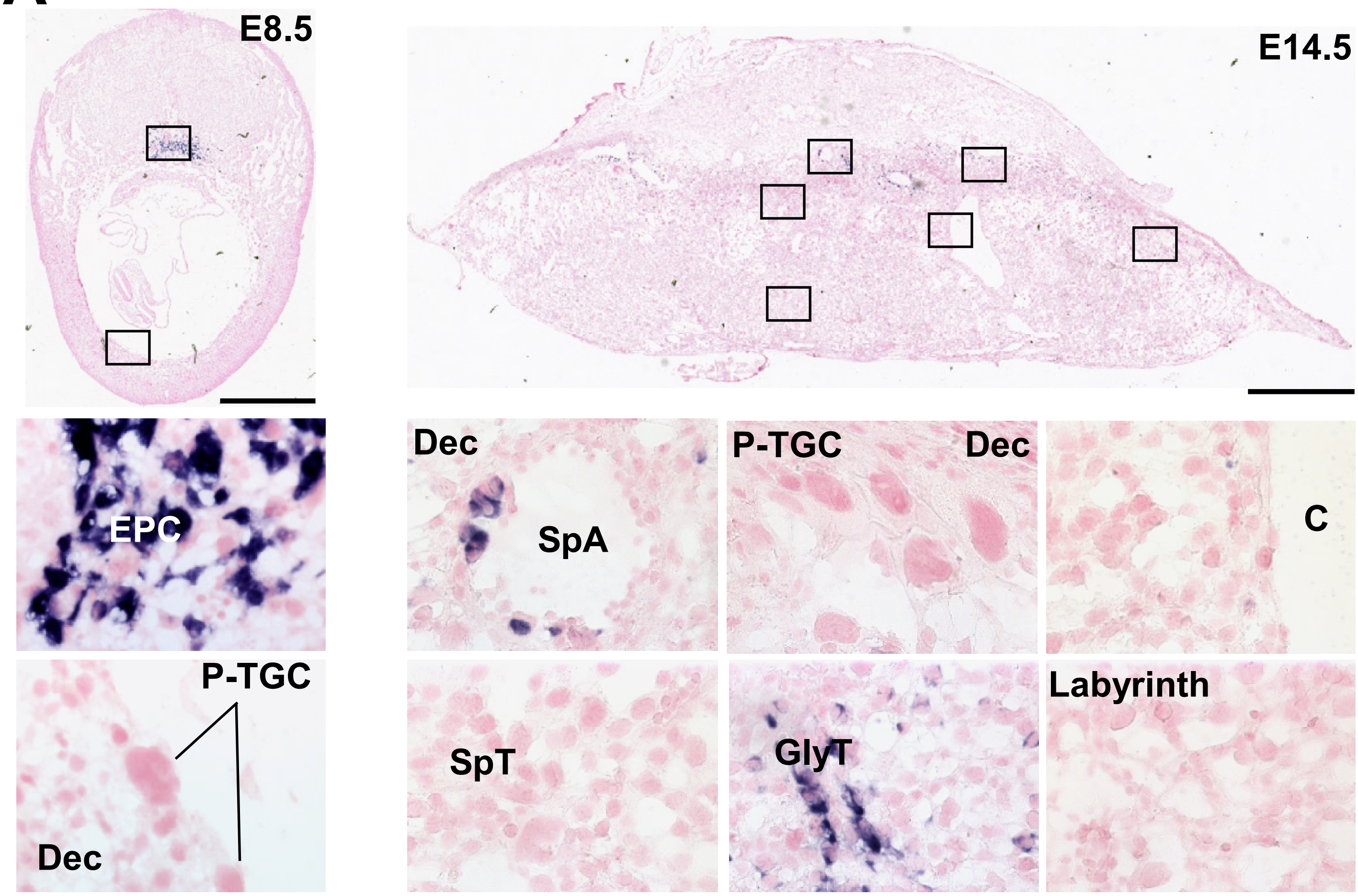

B

Prl7c1

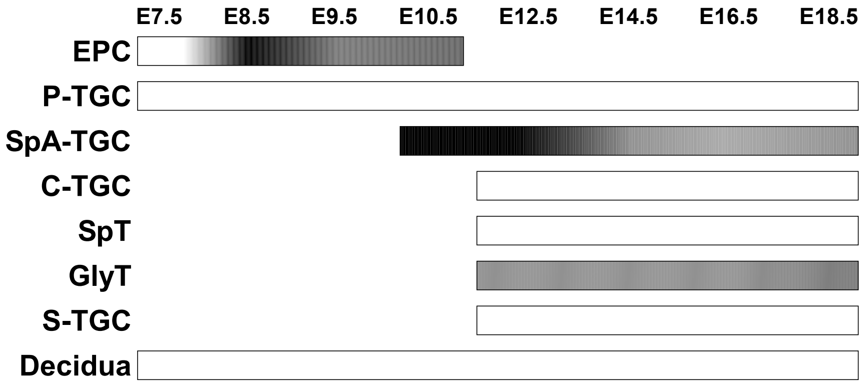

Prl7c1 (*Prlpo*) is expressed in the EPC at E8.5. At E10.5 expression can be seen in SpA-TGCs and GlyT cells surrounding spiral arteries as well as GlyT throughout the decidua and in the SpT layer. Expression of *Prl7c1* is similar to *Prlpn* rather than *Prl6a1*, where more expression is seen in GlyT within the decidua than within the SpT layer. Although, unlike *Prl7b1,* but similar to *Prl6a1*, *Prl7c1* expression is dramatically reduced in the second half of gestation.

Previous publications showing mouse *Prl7c1* expression: (Wiemers et al., 2003).

Notes: in the current study we did not see the robust expression of *Prl7c1* shown by Wiemers et al. In addition, we did not observe *Prl7c1* expression in the S-TGCs of the labyrinth layer. Differences could possibly be explained by differences in in situ hybridization parameters.
