# Supplementary material for: Spatial and temporal expression of the 23 murine Prolactin/Placental Lactogen-related genes is not associated with their position in the locus
Source: BMC Genomics. 2008 Jul 28;9:352. doi: 10.1186/1471-2164-9-352 (PMC2527339; doi:10.1186/1471-2164-9-352)
Supplement: Additional file 18 — A – In situ hybridizations of early (E8.5) and mid to late gestation (E12.5, E14.5, or E18.5) placenta for each member of the PRL/PL family. Higher magnifications emphasize particular trophoblast subtypes including parietal TGCs, spiral artery TGCs, canal TGCs, sinusoidal TGCs, spongiotrophoblast, glycogen trophoblast cells, and decidua. B – Temporal gene expression data (based in situ hybridization signals) for individual placental cell types. Shades of grey depict an estimation of the percentage of each cell type that expresses the gene. White – 0%, Light grey ~25%, Medium Grey ~50%, Dark grey ~75%, Black > 75%. Summary of in situ hybridization data for Prl2a1. [file 1471-2164-9-352-S18.pdf]

Gene: *Prl2a1* (*Prlpm*)

A

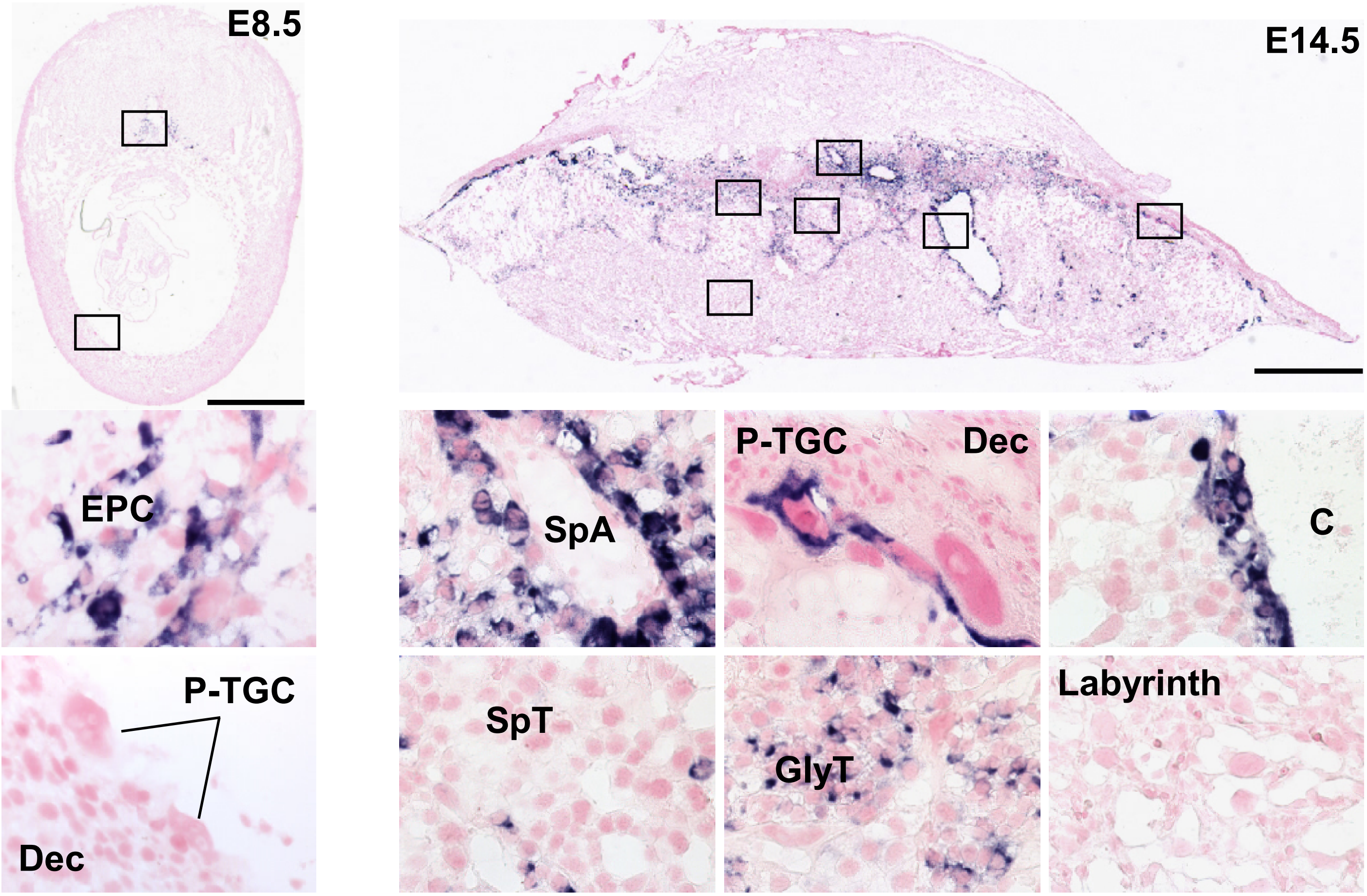

B

Prl2a1

Prl2a1 (*Prlpm*) is initially expressed in the EPC and some P-TGCs at E8.5. P-TGC expression increases and continues throughout gestation. *Prl2a1* expression can also be seen around the spiral arteries after E10.5. C-TGC and GlyT cell expression of *Prl2a1* is evident from E12.5 onward.

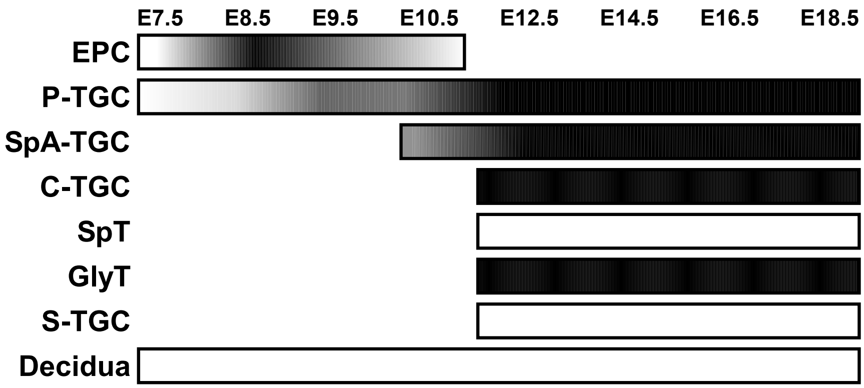

Previous publications showing mouse *Prl2a1* expression: (Dai et al., 2000).
