# Supplementary material for: Spatial and temporal expression of the 23 murine Prolactin/Placental Lactogen-related genes is not associated with their position in the locus
Source: BMC Genomics. 2008 Jul 28;9:352. doi: 10.1186/1471-2164-9-352 (PMC2527339; doi:10.1186/1471-2164-9-352)
Supplement: Additional file 20 — A – In situ hybridizations of early (E8.5) and mid to late gestation (E12.5, E14.5, or E18.5) placenta for each member of the PRL/PL family. Higher magnifications emphasize particular trophoblast subtypes including parietal TGCs, spiral artery TGCs, canal TGCs, sinusoidal TGCs, spongiotrophoblast, glycogen trophoblast cells, and decidua. B – Temporal gene expression data (based in situ hybridization signals) for individual placental cell types. Shades of grey depict an estimation of the percentage of each cell type that expresses the gene. White – 0%, Light grey ~25%, Medium Grey ~50%, Dark grey ~75%, Black > 75%. Summary of in situ hybridization data for Prl4a1. [file 1471-2164-9-352-S20.pdf]

Gene: *Prl4a1* (*Prlpa*)

A

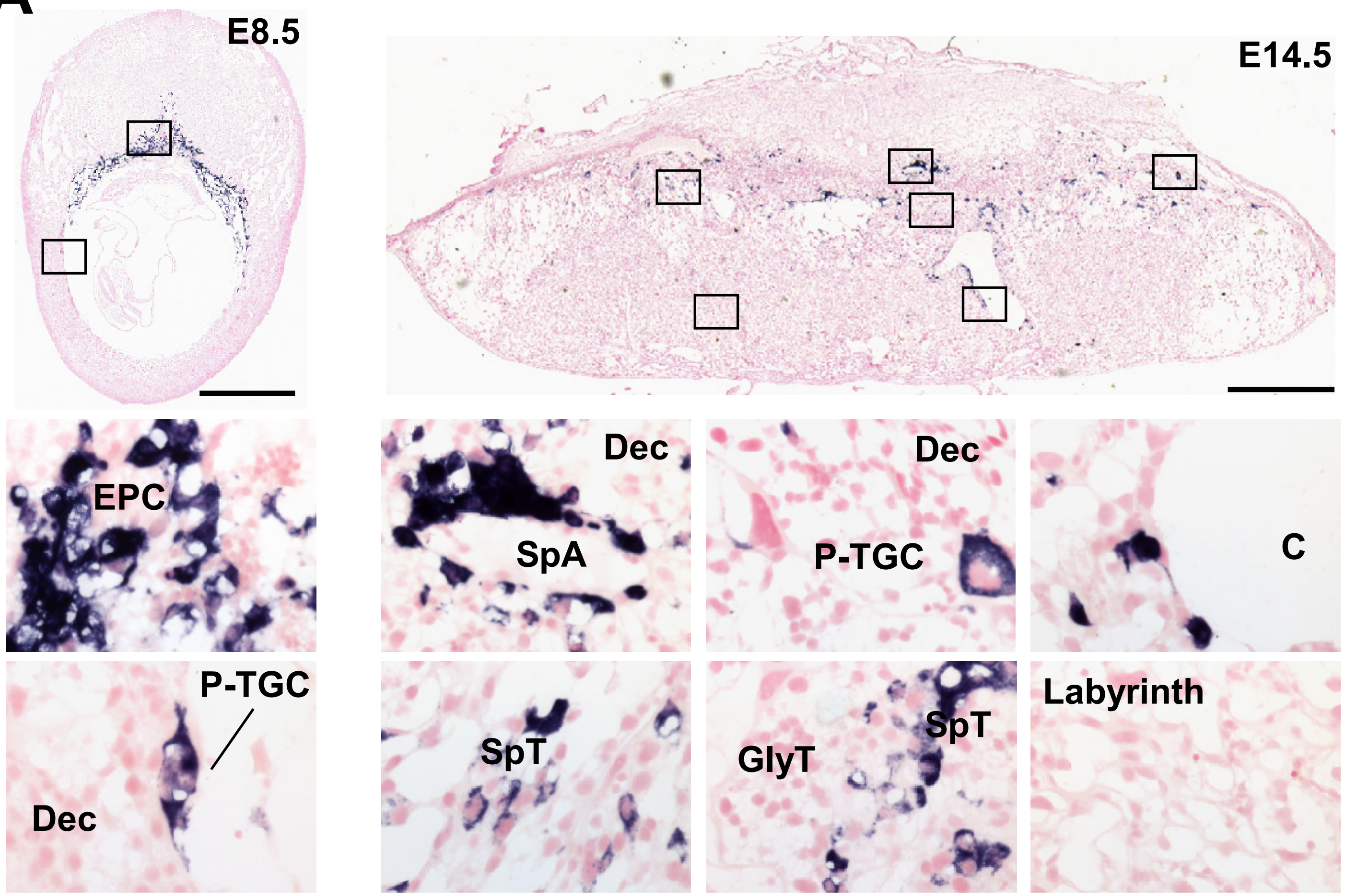

B

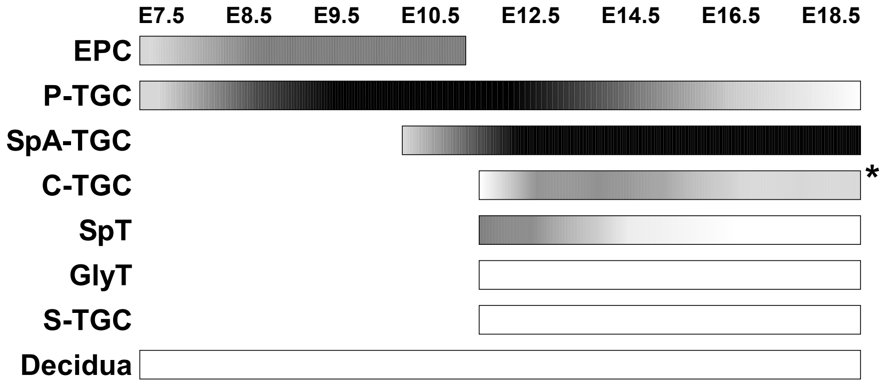

Prl4a1

Prl4a1 (*Prlpa*) is expressed early within the EPC and secondary P-TGCs. In addition, *Prl4a1* is highly expressed in GlyT and/or SpA-TGC surrounding the spiral arteries. As *Prl4a1* expression tapers off in the second half of gestation, expression in and around the spiral arteries remains very high. A subset of SpT cells also express *Prl4a1*, although expression in this subtype is reduced over the course of gestation. Some cells lining the canal spaces, probably C-TGCs but possibly also SpT, express *Prl4a1*, predominantly in the top portion of the canal which lies within the spongiotrophpblast layer. * There is a slight strain difference in *Prl4a1* expression as more C-TGCs appear *Prl4a1* (+) in C57/B6 and 129svj backgrounds than in the CD1 background.

Previous publications showing mouse *Prl4a1* expression:
(Lin et al., 1997a; Muller et al., 1998; Muller et al., 1999; Ma and Linzer, 2000; Ain et al., 2004).
