# Supplementary material for: Spatial and temporal expression of the 23 murine Prolactin/Placental Lactogen-related genes is not associated with their position in the locus
Source: BMC Genomics. 2008 Jul 28;9:352. doi: 10.1186/1471-2164-9-352 (PMC2527339; doi:10.1186/1471-2164-9-352)
Supplement: Additional file 21 — A – In situ hybridizations of early (E8.5) and mid to late gestation (E12.5, E14.5, or E18.5) placenta for each member of the PRL/PL family. Higher magnifications emphasize particular trophoblast subtypes including parietal TGCs, spiral artery TGCs, canal TGCs, sinusoidal TGCs, spongiotrophoblast, glycogen trophoblast cells, and decidua. B – Temporal gene expression data (based in situ hybridization signals) for individual placental cell types. Shades of grey depict an estimation of the percentage of each cell type that expresses the gene. White – 0%, Light grey ~25%, Medium Grey ~50%, Dark grey ~75%, Black > 75%. Summary of in situ hybridization data for Prl5a1. [file 1471-2164-9-352-S21.pdf]

Gene: *Prl5a1* (*Prlpl*)

A

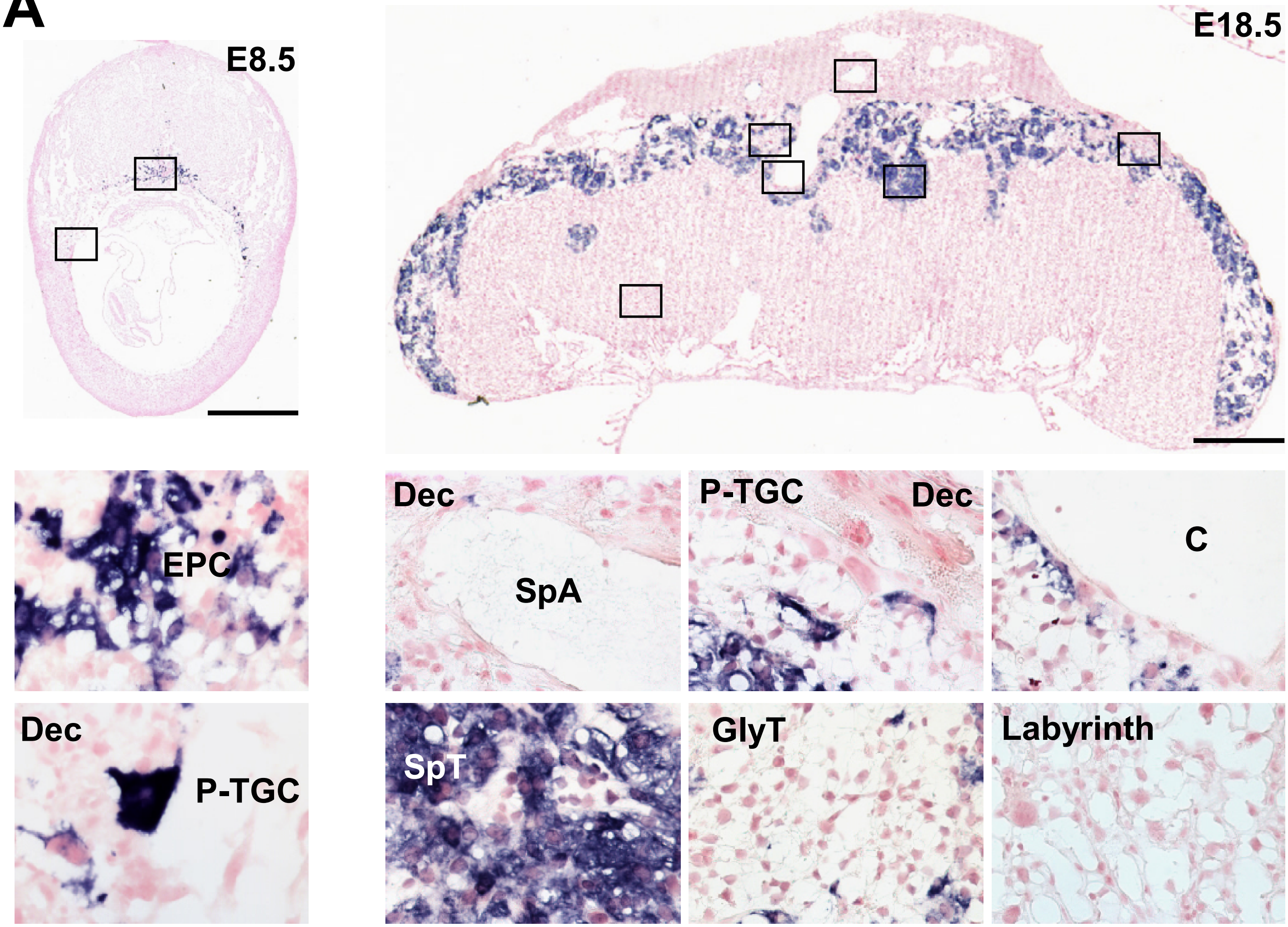

B

Prl5a1

Prl5a1 (*Prlpl*) displays a biphasic expression pattern. Initial expression can be seen in the EPC and outer secondary P-TGCs at E8.5. Expression disappears until later in gestation where SpT cells begin to express *Prl5a1* around E16.5 until term. In addition, sparse expression of *Prl5a1* in SpA-TGCs can occasionally be detected.

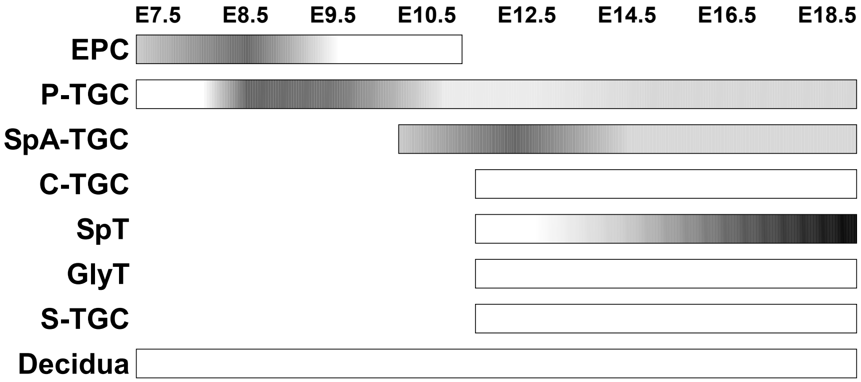

Previous publications showing mouse *Prl5a1* expression: (Wiemers et al., 2003).
