# Supplementary material for: Spatial and temporal expression of the 23 murine Prolactin/Placental Lactogen-related genes is not associated with their position in the locus
Source: BMC Genomics. 2008 Jul 28;9:352. doi: 10.1186/1471-2164-9-352 (PMC2527339; doi:10.1186/1471-2164-9-352)
Supplement: Additional file 19 — A – In situ hybridizations of early (E8.5) and mid to late gestation (E12.5, E14.5, or E18.5) placenta for each member of the PRL/PL family. Higher magnifications emphasize particular trophoblast subtypes including parietal TGCs, spiral artery TGCs, canal TGCs, sinusoidal TGCs, spongiotrophoblast, glycogen trophoblast cells, and decidua. B – Temporal gene expression data (based in situ hybridization signals) for individual placental cell types. Shades of grey depict an estimation of the percentage of each cell type that expresses the gene. White – 0%, Light grey ~25%, Medium Grey ~50%, Dark grey ~75%, Black > 75%. Summary of in situ hybridization data for the Prl2c genes. [file 1471-2164-9-352-S19.pdf]

Genes: *Prl2c2*, *Prl2c3*, *Prl2c4*, *Prl2c5* (*Plf*)

A

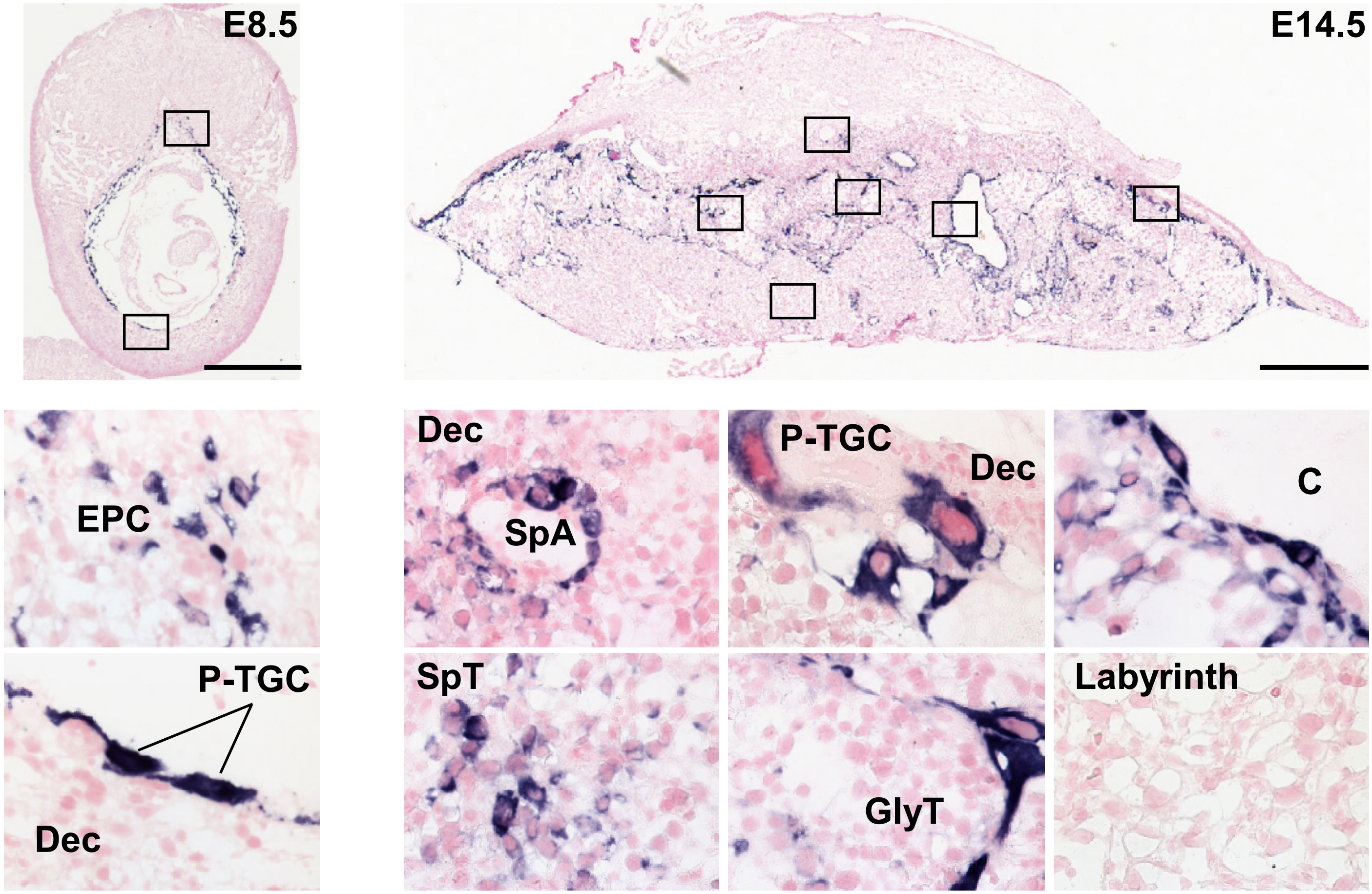

B

Prl2c2-5

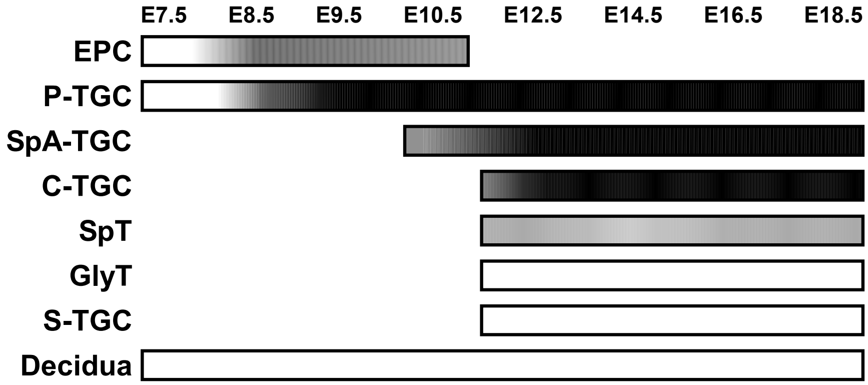

There are 4 annotated *proliferin* (*Plf*) genes, *Prl2c2-5.* However, in situ or northern blot analysis cannot discern between *Prl2c* gene expression. Therefore, all expression data presented here is referred to simply as *Prl2c*, reflecting the unknown origin of expression. *Prl2c* are expressed early in P-TGCs as well as the EPC. It is unclear whether the *Prl2c* (+) cells of the EPC are early differentiating TGCs, TGC precursors or other trophoblast subtype precursors as *Prl2c* is expressed later in gestation in multiple trophoblast subtypes. SpA-TGCs express *Prl2c*, as do C-TGCs. Interestingly, SpT cells express *Prl2c* throughout the second half of gestation, but unlike other prolactin family members expressed by SpT cells, *Prl2c* expression is restricted to the borders of the SpT layer and is mostly absent from the inner SpT cells of the layer.
